# Abiotic Degradation Technologies to Promote Bio-Valorization of Bioplastics

**DOI:** 10.3390/polym17233222

**Published:** 2025-12-03

**Authors:** Karen Gutiérrez-Silva, Natalia Kolcz, Maria C. Arango, Amparo Cháfer, Oscar Gil-Castell, Jose D. Badia-Valiente

**Affiliations:** 1 Research Group in Materials Technology and Sustainability (MATS), Department of Chemical Engineering, School of Engineering, University of Valencia, Av. Universitat s/n, 46100 Burjassot, Spain; karen.gutierrez@uv.es (K.G.-S.); maria.c.arango@uv.es (M.C.A.); amparo.chafer@uv.es (A.C.); 2 Institute of Polymer and Dye Technology, Faculty of Chemistry, Lodz University of Technology, Stefanowskiego 16, 90-924 Lodz, Poland; 3 Agroindustrial Research Group, Department of Chemical Engineering, Universidad Pontificia Bolivariana, Cq. 1 #70-01, Medellín 050031, Colombia

**Keywords:** bioplastics, valorization, biodegradation, abiotic degradation, pretreatments

## Abstract

Biodegradable bioplastics have emerged as a promising sustainable alternative to minimize the environmental impact of traditional plastics. Nevertheless, many of them degrade slowly under natural or industrial conditions, raising concerns about their practical biodegradability. This fact is related to the high-order structure of the polymer backbones, i.e., high molar mass and high crystallinity. Research efforts are being devoted to the development of technologies capable of reducing the length of polymer segments by accelerated chain scission, which could help improve biodegradation rates upon disposal of bioplastic products. The objective of this review is to examine the current state of the art of abiotic degradation techniques, physically driven by temperature, mechanical stress, UV/gamma/microwave irradiation, or plasma or dielectric barrier discharge, and chemically induced by ozone, water, or acidic/basic solutions, with the aim of enhancing the subsequent biodegradation of bioplastics in controlled valorization scenarios such as composting and anaerobic digestors. Particular attention is given to pretreatment degradation technologies that modify surface properties to enhance microbial adhesion and enzymatic activity. Technologies such as ozonation and plasma-driven treatments increase surface hydrophilicity and introduce functional groups with oxygen bonds, facilitating subsequent microbial colonization and biodegradation. Irradiation-based techniques directly alter the chemical bonds at the polymer surface, promoting the formation of free radicals, chain scission, and crosslinking, thereby modifying the polymer structure. Pretreatments involving immersion in aqueous solutions may induce solution sorption and diffusion, together with hydrolytic chain breakage in bulk, with a relevant contribution to the ulterior biodegradation performance. By promoting abiotic degradation and increasing the accessibility of biopolymers to microbial systems, these pretreatment strategies can offer effective tools to enhance biodegradation and, therefore, the end-of-life management of bioplastics, supporting the transition toward sustainable cradle-to-cradle pathways within a biocircular economy.

## 1. Overview of Bioplastics: Concepts and Properties

Plastic refers to products consisting of natural or synthetic chemical compounds primarily made from polymers formed through the polymerization of monomers [1]. Thermoplastics, in contrast to thermosets, can be remolded under heat and pressure, which offers recyclability. Traditional petroleum-based thermoplastics, such as polyethylene (PE), polypropylene (PP), poly(ethylene terephthalate) (PET), poly(vinyl chloride) (PVC), polystyrene (PS), and polyamide (PA), are commodities for widely used applications such as packaging, automotive manufacturing, agriculture, and textiles [2]. They offer advantageous properties such as good mechanical strength, effective gas barriers, and low production costs, making them suitable for a wide variety of applications. However, their conventional production, largely reliant on fossil resources, and their non-biodegradable nature upon disposal lead to serious environmental challenges, including a reduction in non-renewable resources and the accumulation of persistent waste in landfills [3,4]. Even though the degradation of plastics is feasible in the natural environment, the lifespan of plastics can extend from 10 to 600 years [4,5], depending on their chemical structure, additive content, environmental exposure conditions (such as temperature, humidity, UV radiation, and microbial activity), and the specific disposal scenario (e.g., landfill, marine, or soil environments).

**Figure 1:**
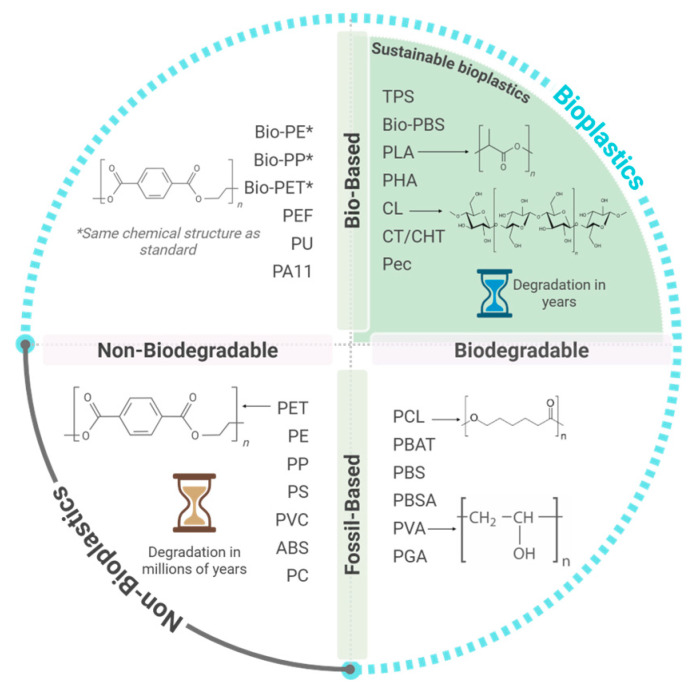
Classification of polymers and biopolymers based on the origin of monomers and their environmental degradability. Adapted from [11].

Bioplastics have emerged as an alternative to traditional plastics. They are a diverse family of polymer materials, which are either bio-based, biodegradable, or feature both properties [6]. When polymers are obtained, either partially or fully, from renewable biological sources (e.g., corn, sugarcane, or wood), they are considered bio-based [7]. Biodegradable polymers are defined as materials where the degradation process occurs through the breakdown of their macromolecular backbone into oligomeric or monomeric species and their subsequent assimilation by microorganisms to convert them into water, carbon dioxide, or methane and biomass [6,8]. The biodegradability is not inherently linked to a polymer’s source, where a biodegradable polymer can be derived from bio- or fossil-based feedstocks, with the biodegradation rate depending on both the chemical composition and the material’s structural properties, mainly crystallinity and molar mass, and on the environmental conditions in which it takes place [9]. In this context, bioplastics can be bio-based without being biodegradable, fossil-based yet biodegradable, or both bio-based and biodegradable. Figure 1 illustrates this classification, where fossil-based and non-biodegradable polymers are excluded from the bioplastic category [10].

In recent years, particular attention has been devoted to developing and investigating fully biodegradable bioplastics with tunable physicochemical properties, enabling their use in the manufacture of diverse products for a wide range of applications [12,13,14]. Some of the thermo-mechanical properties of the most common biodegradable bioplastics are summarized in Table 1.

As a natural consequence of progress in this field, bioplastic production capacities have increased, currently accounting for around 0.7% of the total global plastic production, which exceeded 413 million tons annually in 2023 [19]. The overall bioplastic production reached around 2 million tons in 2023, driven by increasing demand and the development of more advanced applications and products, with projections estimating that this will rise to 5.73 million tons by 2029 [19]. From these, it is estimated that approximately 66% of global bioplastic production capacity will correspond to materials that are both bio-based and biodegradable. For instance, polylactic acid (PLA) is expected to account for 42.3% of the production, while polyhydroxyalkanoates (PHAs) will represent around 17.0% [19]. Among the 54 million tons of plastics produced in Europe in 2023, 1.4% were bio-based and bio-attributed, representing an increasing tendency in circular plastic production [20,21].

Like traditional polymers, bioplastics also become waste at the end of their service life. Some studies have been devoted to the mechanical recycling of bioplastics to extend the material cycle before discarding [22,23,24,25]. Efforts to increase the lifespan of plastics are reflected in the global rise of mechanical recycling, which reached 8.7% of total plastic production in 2023 [20]. However, multiple thermo-mechanical cycles can reduce molar mass, reducing functional properties and therefore downgrading the product [26,27,28].

Currently, efforts to manage these wastes at an industrial scale focus on advanced recycling and bio-recovery methods to improve environmental performance and reduce landfilling and GHG emissions. Although these materials offer the advantage of being potentially biodegradable and compostable, their large-scale integration into current recycling facilities poses considerable challenges.

Among the most critical issues are the difficulty of separating bioplastics from conventional polymers, the relatively low proportion of bioplastics in waste streams, and the limitations of infrastructure primarily designed for mechanical recycling. These factors hinder both the sufficient accumulation of material and its efficient treatment.

Nevertheless, the scalability potential of biological recycling is significant, provided that more accurate sorting technologies are developed, differentiated collection policies are promoted, and waste management systems are adapted [29]. In this context, it is essential to assess the opportunities and barriers that will determine the feasibility of biological recycling of bioplastics as an integral component of a circular economy.

Once separation concerns have been overcome, the other main issue is the variability in the biodegradation rate of bioplastics, as it closely depends on the environment in which they develop (soil, water, marine, or compost), with multiple mechanisms influenced by external factors that may act simultaneously to promote degradation with potential synergistic returns. For instance, common biopolymers such as PLA, PHB, PBS, and PCL often exhibit different degradation rates [30], depending on the standard test method used and the environmental conditions involved, given their dissimilar physico-chemical structure and properties. Some examples of biodegradation are given for bioplastics herein.

For PLA, the biogas production from the anaerobic digestion of PLA cups achieved 564 L biogas kg^−1^ vs. in 280 days (a 66% biodegradation yield) under mesophilic conditions, making it unacceptable at a technical scale [31]. In addition, anaerobic sludge from wastewater treatment has also been employed as a complex microbial environment to study biopolymer degradation of PLA and PBAT, with a very low biodegradation rate achieved after 20 days of exposure [32].

Within the framework of industrial controlled waste valorization facilities, processes such as composting need a specific residence time to ensure proper waste treatment, which cannot guarantee total valorization of bioplastics [9]. In aerobic environments, when PLA is subjected to the conditions of ISO 14855, it disintegrates after 90 days of exposure to the simulated industrial composting environment [33], although it has a low biodegradation rate [34,35]. Bioplastics marketed as compostable with certifications according to EN13432 fail to pass the 90% threshold [33].

Even more worrying, given the non-controlled disposal of biodegradable bioplastics, they may take years to fully degrade on land or in seawater because the eventual surrounding conditions, such as temperature, humidity, and microorganisms’ presence, may not correspond to the material properties to induce effective degradation [36]. PLA and PBAT may show limited biodegradation in soil [37,38,39] and aquatic environments [40], highlighting why their disposal in nature, home composters, or organic waste streams is not recommended [41]. Other studies determined that after 18 months of burial in soil, biodegradation of mulch films of PLA and PBAT achieved 46% and 88.1%, respectively, with a six-times-higher microplastic (MP) content for PBAT than for PLA [42]. While PBAT degradation is associated with a higher short-term release of MPs, PLA degradation tends to stimulate microbial respiration, emitting more CO_2_ and depleting native soil organic carbon, both representing undesirable environmental side effects [42]. Even under industrial composting conditions, PBAT-based carrier bags disintegrated to 95% of the initial mass in 12 weeks, with some particles with a size above 2 mm. Further analysis revealed some undesirable decomposition products, such as acid butanediol esters still present in the composting media after one year [43]. As a result, a substantial amount of bioplastic waste accumulates due to the lack of an effective disposal solution, perpetuating plastic pollution.

Therefore, the understanding of the relationship between material properties and bio-valorization routes, together with the development of methods to accelerate the biodegradation of biopolymers, is of high relevance. Several reviews have addressed topics related to polymer valorization from different perspectives. Some have summarized the microorganisms and enzymes capable of degrading plastics [44], while others have focused on the factors governing biodegradation processes, including both biotic and abiotic perspectives [41]. More recently, the use of abiotic factors as pretreatment strategies to promote the degradation of plastics has gained attention [9,45,46].

However, within the existing literature, most reviews focus on commodity plastics rather than on biodegradable bioplastics, and they rarely report metrics showing how abiotic modifications enhance biodegradation under composting or anaerobic conditions [47,48]. As summarized in Table 2, key dimensions of bioplastic degradation are contrasted across existing reviews, using a qualitative scoring system to indicate how extensively each topic has been covered. This comparison shows that most reviews focus on plastic commodities, treat these aspects separately, and rarely connect pretreatment strategies with their integration into real waste management scenarios. Consequently, an integrated perspective that links pretreatments, relevant degradation metrics, and downstream valorization is still lacking.

The present review aims to address this gap by integrating two complementary aspects. First, it consolidates the available evidence on how abiotic pretreatments modify bioplastic properties in ways that influence their subsequent biodegradation and valorization. Second, it identifies the most informative indicators to track the pretreatment-to-biodegradation pathway, highlighting cases where a direct causal link has been experimentally demonstrated. Through this dual focus, this review aims to offer an updated overview of abiotic degradation technologies applied to bioplastics, focusing on their role in enhancing subsequent biological valorization routes towards cradle-to-cradle pathways under a biocircular economy framework.

## 2. Bio-Valorization of Biodegradable Bioplastics

### 2.1. Degradation of Bioplastics

The term *degradation* is broadly used to encompass all processes that alter the properties and overall performance of polymers, spanning from synthesis to end of life [41]. More specifically, polymer degradation refers to an irreversible change in chemical structure through the fragmentation of macromolecules into low-molar mass chains or segments, resulting in a modification of their physical properties, such as discoloration, brightness, and loss of mechanical resistance properties, as well as chemical modifications, including the disappearance, generation, and transformation of functional chemical groups [41,49]. For the sake of context, this review is focused on the biodegradation of biopolymer-based waste upon disposal across three environments: soil, compost, and anaerobic digestion, mainly under controlled conditions.

Traditional classification of degradation pathways refers to the process occurring in the absence (abiotic) or presence (biotic) of biological activity. Abiotic degradation includes physical and chemical degradation mechanisms such as mechanical, thermal/thermo-oxidative, photo/photo-oxidative, hydrolytic (chemical), and ozonation degradation, which environmental factors like oxygen, moisture, heat, and ultraviolet radiation can trigger [11,50]. In contrast, biotic degradation involves the enzymatic or microbial breakdown of polymers into water, carbon dioxide/methane, and biomass. These pathways can act independently or synergistically, with abiotic processes often facilitating subsequent microbial attack by reducing molar mass or introducing polar functional groups that allow subsequent reaction and chain scission [51]. With an understanding of the mechanisms that are involved, the biodegradation process can be optimized [8]. In the literature, some polymer degradation pathways, such as biodegradation, photodegradation, and thermo-oxidative degradation, are thoroughly discussed [3,50,52,53].

### 2.2. Biodegradation Strategies for Bioplastics

Within the broad biodegradation framework, biological valorization refers to the process where, in controlled conditions, plastic compounds undergo a biochemical transformation in mineralization into biomass and gases, primarily carbon dioxide (CO_2_) under aerobic conditions or methane (CH_4_) under anaerobic conditions. Table 3 summarizes the main differences between aerobic and anaerobic pathways, highlighting the influence of the oxidative environment on the degradation performance, end products expected, and environmental implications [7,50,54,55].

The core of biological valorization of bioplastics in natural environments relies on the degradation activity of microorganisms, where enzymatic processes driven by living cells are essential. In contrast, enzymatic degradation refers to the in vitro activity of isolated enzymes acting directly on the polymer substrate [1,45]. During biological valorization, several stages happen in sequential steps [48,51]:

(1) Abiotic degradation is to a certain extent considered the initial step in the biodegradation process, where external factors such as sunlight, heat, oxygen, and moisture trigger thermal, chemical, mechanical, and photodegradation pathways. This stage weakens the polymer structure, even producing an initial fragmentation [8].
(2) Subsequently, biodeterioration occurs, characterized by the colonization of the polymer surface by microorganisms, thanks to biofilm formation. The microorganism initially attaches to the polymer surface via the cell pole or flagellum, with irreversible attachment happening through a glue-like substance and tail-like structures. This is followed by the secretion of extracellular polymeric substances (EPSs), which constitute a slimy matrix composed of proteins, polysaccharides, lipids, and nucleic acids [56]. EPSs promote the formation of multicellular clusters that maintain close contact with both the substrate and neighboring cells, establishing a stable biofilm that may support subsequent enzymatic activity and biodegradation under favorable conditions.
(3) As polymers are insoluble and too large for direct microbial uptake, extracellular enzymatic activity is essential in the depolymerization stage **[57]**. Microorganisms secrete extracellular enzymes that cleave the chemical bonds within polymer chains. These enzymes act as biological catalysts, significantly accelerating reactions that would otherwise proceed very slowly, even for bioplastics derived from renewable resources, such as PLA and PHAs [58]. The potential of purified enzymes, such as lipases, cutinases, and proteinase K, has been extensively studied [59,60], particularly in controlled systems, where they have demonstrated high efficiency in catalyzing the cleavage of ester bonds in bio-polyesters. This enzymatic depolymerization transforms high-molar mass macromolecules into smaller units as oligomers, dimers, and monomers [58,61].
(4) Monomers surrounding the microbial cells pass through the cellular membrane to be bioassimilated. Their uptake can occur easily thanks to passive diffusion [62] or through specific membrane carriers [51]. Monomers that cannot easily be transported into cells can undergo biotransformation reactions, giving intermediate compounds that may or may not be further assimilated [51].
(5) Once the transported monomers are inside cells, they are oxidized to adenosine triphosphate (ATP) through three mechanisms: aerobic respiration, anaerobic respiration, and fermentation, depending on microbial capacities to grow in the presence or absence of oxygen. As a result of catabolic activity, monomers are mineralized into CO_2_, methane, nitrogen, and water [63].

Within the existing waste management system, biological valorization primarily occurs through two controlled routes: in composting systems, which rely on aerobic microbial activity at elevated temperatures, and in anaerobic digestion systems, which operate in oxygen-free environments to recover energy as methane-rich biogas [64].

The process of biodegradation within composting conditions serves as a widely adopted approach for managing organic waste like natural materials, food scraps, and yard waste as they reach the end of their life cycle. Typically, industrial composting is performed under thermophilic (~55 to 65 °C) conditions, with a moist environment (~50 to 60%) and adequate aeration. Microorganisms use organic material as carbon and energy sources to principally produce carbon dioxide, water, and stabilized compost as the end product [65]. In this form, composting leads to biological stabilization of the organic waste, yielding a stable material that can be repurposed for agricultural activities at a large scale, localized urban projects on a smaller scale, and even personal gardening at home [9]. Comparing industrial and home composting, industrial composting is generally preferred because biodegradation proceeds more efficiently under controlled conditions. Additionally, higher biodegradation rates are achieved since elevated moisture content, aeration, and temperature favor the activity of thermophilic microorganisms. In contrast, in home composting, mesophilic microbes are responsible for the hydrolytic degradation process [66,67]. Anaerobic valorization strategies include anaerobic digestion, which occurs in oxygen-deprived reactors in both mesophilic (37 °C) and thermophilic (55 °C) conditions in biogas plants. As a result, methane gas, carbon dioxide, water, H_2_S, and ammonia are the main by-products [8,65]. The biodegradation reactions under aerobic and anaerobic conditions can be described as follows in Equations (1) and (2) [8,65,68]:

(1) $$C_{polymer}+\;O_{2}\;\to \;{CO}_{2}+\;H_{2}O+\;C_{biomass}+Energy$$

(2) $$C_{polymer}+H_{2}O+Nutrients\;\to {CO}_{2}+{CH}_{4}+{NH}_{3}+H_{2}S+Energy$$

### 2.3. Standards for Aerobic and Anaerobic Biodegradation and Disintegration Testing

Standards are essential instruments to ensure that the features of bioplastics reported in fact sheets are benchmarked under the same controlled tests. The standardization scenario distinguishes between (i) certifications for products, as ISO 13432 pass criteria for a product to be marketable as compostable, and (ii) laboratory test methods to quantify biodegradation-specific indicators via mineralization of organic carbon or physical disintegration.

In the first scenario, internationally recognized standards play a key role in defining the biodegradability and compostability criteria that bioplastics must meet to be properly classified and commercialized. The primary standardization organizations in this field are ASTM (American Society for Testing and Materials), ISO (International Organization for Standardization), and CEN (European Committee for Standardization).

For bioplastics, the frequently reported testing procedures are related to aerobic degradation at an industrial compostable scale [10]. Among them, the most widely adopted standards are as follows:

1. EN 13432: In the European marketplace, a packaging product must meet these minimum requirements to be marketable as compostable and thus be processed by industrial composting during end of life [69]: disintegration of ≥90% of the mass that passes through a 2 mm sieve after 12 weeks, biodegradation of ≥90% of the organic carbon to be mineralized into CO_2_ within ≤6 months, absence of negative effects on the composting process, and heavy metal quantities below the given maximum values (absence of ecotoxicological effects) [70].
2. ASTM D6400: This contains the framework for the United States of America and specifies a minimum of 60% biodegradation for heteropolymers and 90% for homopolymers within 180 days at ≥60 °C to be certified as compostable. The test method is equivalent to ISO 17088 [71].
3. ISO 17088: This distinguishes requirements for labelling plastic products as biodegradable during composting, compostable, or compostable in municipal and industrial composting facilities. For the compostable requirements, it demands that 90% of the organic carbon is converted to CO_2_ within 180 days, relative to a cellulose reference. As well as EN 13432, ISO 17088 does not strictly specify conditions, though it states that composting must be developed in well-managed industrial composting processes [72]. However, this standard refers to the typical tests applied by ISO 14855, where biopolymers classified as compostable must biodegrade at thermophilic conditions (58 ± 2 °C) at 55% relative humidity [73].

Following the standards that provide laboratory-scale methods to evaluate the degree of degradation of plastic materials, some simulate industrial conditions. Among them, some are focused on mineralization, such as monitoring the CO_2_ evolution in ISO 14855 and the biogas evolution in ISO 15985 [73]. Others, such as ISO 20200, assess the physical breakdown of a plastic sample when exposed to controlled aerobic composting at temperatures around 58 °C. In this scenario, a material is considered disintegrated if more than 90% of its initial mass passes through a 2 mm sieve at the end of the test [74]. A summary of the pass criteria for the commonly used testing methods at laboratory scale can be found in Table 4.

For anaerobic testing conditions, not only ISO 15985 but also standards such as ISO 14853 and ISO 13975 determine the biodegradation of a polymeric substrate in a laboratory environment by the analysis of released biogas [75,76,77]. However, the mentioned standards do not completely reproduce the normal conditions that are found in a biogas plant, and no pass criteria are specified in terms of mineralization rates to consider that a material is biodegraded [78]. The Biochemical Methane Potential (BMP) test is commonly used because it operationalizes anaerobic biodegradation by measuring methane and CO_2_ production and reporting comparable metrics [79]. The report of the specific methane yield, the fraction of the theoretical methane potential, and kinetic predictors provide a quantitative basis to infer degradation in digesters, even in the absence of formal pass/fail mineralization criteria.

Also, marine (ASTM D6691 [80]), freshwater (ISO 14851 [81]), and aquatic anaerobic digestion (ISO 11734 [82]) and soil (ISO 17556 [83]) simulated environments may completely degrade bioplastics [84]. Nevertheless, the non-strict control of degradative parameters, such as temperature, humidity, radiation, and microbiota, presents industrial limitations. While unmanaged environments provide conditions closer to those in which plastic degradation naturally occurs, the variability and limited reproducibility of results restrict their integration into standardized testing frameworks.

### 2.4. Factors Affecting Bioplastic Biodegradation

Biodegradation of bioplastics depends on multiple factors, and slow or incomplete mineralization in a given setting may reflect environmental limitations rather than an intrinsic lack of biodegradability. These factors that influence degradation can be broadly categorized into intrinsic factors, inherent to chemical and structural characteristics of the polymer, and extrinsic factors, mainly associated with the environmental conditions to which the material is exposed during its service life [49,54,55,85,86]. From an abiotic perspective, given the aim of this review, the main factors are exposed in the next sub-sections.

#### 2.4.1. Factors Inherent to the Polymer

The chemical structure of the polymer backbone strongly influences the degradation rate of bioplastics [49]. In particular, bond dissociation energies (BDEs) determine the susceptibility of specific bonds to cleavage. Typical average energies for homolytic cleavage of chemical bonds such as C-C, C-H, C-O, C=C, and O-H range from ~80 kcal/mol up to 160 kcal/mol, although the chemical environment can modify the effective energy required for cleavage [87]. These values typically refer to processes initiated by photo-oxidative or thermo-oxidative mechanisms. Polymers with lower BDE bonds are more prone to chain cleavage and subsequent oxidation.

The presence of specific functional groups, such as esters in polyesters, can increase the susceptibility of polymers to degradation by both abiotic (e.g., hydrolysis) and biotic (e.g., enzymatic hydrolysis) pathways [88]. In the case of hydrolytic degradation, where heterolytic cleavage of ester linkages (C-O bonds) occurs, reported activation energies are significantly lower; for instance, for PLA, values are in the range of 9–13 kcal/mol, depending on the surrounding medium and the autocatalytic nature of the process [89].

From the chain structure point of view, intrinsic factors such as number and weight-average molar mass (*M_n_* and *M_w_*), polydispersity index (PDI), amorphous and crystalline zone distribution, crosslinking density, and tacticity determine how accessible and susceptible the degradative agents are to the chemical bonds. Polymers with a lower molar mass are more prone to microbial degradation, as they are more likely to depolymerize into oligomers and monomers [4]. Consequently, high-molar mass biopolymers may degrade faster when abiotic chain scission reduces *M_n_* before biodegradation [8]. Molar mass controls degradation because it determines chain-end density and fragment solubility. Reduction in *M_n_* increases the number of reactive end groups (e.g., –COOH/–OH in polyesters) and yields smaller, more soluble fragments. Under random chain scission, *M_n_* decreases faster than *M_w_*, so PDI typically increases in early stages, reflecting a broader distribution enriched in low-molar mass species. In contrast, crosslinking limits accessibility of the polymer chains to microbial attack [90].

The degree of crystallinity is another crucial factor that affects biotic and abiotic degradation [7,91]. In the early stages of degradation, the amorphous regions are degraded preferentially over the crystalline ones. This occurs because the amorphous phase, characterized by a less ordered molecular arrangement, is more accessible and therefore more susceptible to attack by both abiotic and biotic factors than the crystalline domains [16]. For example, in biocopolymers such as PBSA, the low degree of crystallinity results from the higher content of butylene adipate (BA) segments in the copolymer, which accelerates biodegradation in soil environments [92]. In contrast, high crystalline order and thus low accessibility to microorganisms’ activity reveal a lower biodegradation rate [40].

Among surface properties, hydrophilicity and roughness are especially relevant. Increasing wettability promotes water uptake and hydrolysis, and moderate roughness provides sites for biofilm and enzyme attachment, together enhancing biodegradation [93,94].

In summary, more than being of fossil or bio-based origin, the biodegradation and subsequent valorization of biopolymers is deeply dependent on both the chemical nature of the polymer and its physical properties, such as building-block structure, hydrophilicity/hydrophobicity, crystallinity, molar mass, and quantity of labile chemical groups (e.g., ester or ether groups) [7,8,95]. Low molar mass, low crystallinity, high hydrophilicity, and the presence of ester groups help promote the accessibility of microorganisms, microbial adhesion, and the effectiveness of their enzymes for complete valorization [9,45,91].

#### 2.4.2. External Factors

Several investigations have highlighted the strong influence of environmental conditions on the stability and degradation of bioplastics. Factors such as temperature, humidity, light exposure, pH, and oxygen availability can affect both the structural integrity of biopolymers [96,97,98,99,100,101,102] and modulate the efficiency of biodegradation [54,103].

Temperature is an environmental factor that enhances abiotic processes and biotic activity during biodegradation. Reaction rates generally follow Arrhenius kinetics, with an increase in temperature accelerating hydrolysis and thermo-/photo-oxidation reactions, raising chain mobility, and enhancing diffusion of water and oxygen into the polymer matrix. As traditional polymers, biopolymers exhibit a wide range of thermal properties, reflected in temperatures such as glass transition (*T_g_*) and melting (*T_m_*) temperatures, which determine the effectiveness of polymer degradation, due to the influence of the organization of the molecular framework, changing the posterior accessibility to microorganisms and enzymatic activity. Specifically, temperature plays a key role in causing different structural transformations in polymers, depending on the level of thermal energy and time exposure [104]:

− At temperatures below *T_g_*, physical aging occurs, characterized by molecular rearrangement without chemical degradation.
− In the range between *T_g_* and *T_m_*, dimensional stability is compromised and phenomena such as shape loss, recrystallization, and thermal decomposition of low-molar mass additives can be observed.
− When the temperature exceeds *T_m_*, loss of structure of the crystalline region happens, leading to a disorganized melt and collapse of the polymer structure.
− At temperatures above the decomposition temperature (*T_d_*), combustion takes place and the energy stored by the material may be recovered in heat form.

In conclusion, at temperatures between *T_g_* and *T_m,_* the rubbery state of the polymers favors degradation. The segmental mobility and free volume boost microbial activity and water and oxygen diffusion, while loss of dimensional stability and crystalline zone rearrangement may occur [104]. The optimum temperature for polyesters was found to be close to but not to exceed *T_m_* [105].

Complementarily, the temperature of the biodegradation medium modulates the biodegradation rate by affecting both reaction kinetics and microbial/enzymatic activity. The biodegradation rate normally increases in thermophilic conditions. Soil-burial tests at mesophilic conditions (20–28 °C), closely reflecting real environmental soil temperatures, may take hundreds of days to achieve satisfactory biodegradation performance. For instance, PBAT–starch-based pellets (e.g., Mater-bi) reached acceptable mineralization rates, according to EN 17033 standards, only after more than 400 days [85]. However, raising the temperature to hyperthermophilic values may compromise the integrity of the microorganisms [106].

Among environmental factors, solar radiation is the primary driver of natural weathering. The ground-level spectrum extends from ~290 to 3000 nm, and its UV band strongly affects polymer stability. Under outdoor service conditions, bioplastics are principally exposed to ultraviolet UV-A radiation (400 to 315 nm, 3.1–3.9 eV), UV-B radiation (315 to 280 nm, 3.9–4.4 eV), and visible light [49], whereas artificial UV-C radiation (280–100 nm, 4.4–12.4 eV) may also be applied [107]. All these UV radiation types have sufficient energy to break certain chemical bonds in polymers [93], thereby contributing to their partial photodegradation.

Moisture plays a dual role in the degradation behavior of bioplastics. In abiotic processes, the presence of water molecules promotes hydrolysis of labile bonds, particularly ester and ether linkages [51]. In polyesters with aliphatic segments such as PLA and PBAT, the hydrolytic cleavage results in the formation of new hydroxyl and carboxylic groups, leading to a reduction in molar mass [108]. The rate and extent of this process depend on the crystallinity of the polymer, thickness, and water diffusion coefficient, as hydrolysis preferentially takes place in amorphous domains where molecular mobility and water penetration are higher [94,109]. From a biological perspective, humidity is essential to sustain microbial activity and enzyme mobility, thereby facilitating biodegradation.

In parallel, the pH of the hydrolytic medium modulates reactions through catalytic pathways. In ester hydrolysis, the effect of acid or basic solutions can accelerate abiotic degradation by altering both the reaction mechanism and the reaction kinetics [89,110,111,112]. As degradation progresses, changes in the pH can occur. As a result of degradation, monomers and oligomers are released with a pH-dependent nature.

### 2.5. Evidence of Biopolymer Degradation

As previously mentioned, there are several changes biopolymers may suffer when exposed to degrading conditions. These alterations provide measurable evidence of degradation and can be monitored through different methodologies and analytical techniques [41,51,113,114]. The principal methods selected by researchers as tools for characterizing abiotic degradation are grouped in the following subsections.

#### 2.5.1. Macroscopic Variations

Visual examination enables the determination of changes in color, transparency, and size. Obtaining photographs and performing colorimetry studies are common strategies that provide relatable information about appearance, color, and observed macroscopic changes [91]. Yellowing index and CIELAB parameters (b* and L*) are often used to assess color changes in polymers [100,115,116,117].

Complementarily, mass loss reduction measurement is the widest, simplest, and most practical method used by researchers to quantify degradative changes from a macroscopic perspective. Using an analytical balance, mass loss (∆m) is determined by measuring the difference between the initial mass ($m_{o}$) and the final dry mass ($m_{f}$) (Equation (3)).

(3) $$∆m\left(\%\right)=\frac{m_{o}-m_{f}}{m_{o}}\times 100$$

The extent of mass loss depends on the degradative medium [99], and its decrease does not ensure eventual absorption by microorganisms [118], highlighting its limitations and the necessity of complementing its measurement with other analytical methods.

#### 2.5.2. Microscopic Morphology and Surface Properties

Microscopic surface observation is commonly used to elucidate changes induced by biotic and abiotic degradation [86]. Scanning electron microscopy (SEM) enables the visualization of surface deterioration, fragmentation, and surface roughness variation as a consequence of degradation processes. Also, the detection of microorganisms’ colonization can be pursued with SEM, together with optical microscopy [43,119,120].

Polarized optical microscopy (POM) can give extra information by helping to elucidate the variation in the morphology of spherulites. Changes in crystallization and an increase in crystal domain orientation during biodegradation in seawater were used to monitor the biodegradation of PLA, PCL, and PHB [40].

Complementarily, surface changes in polymer properties are generally evaluated through the study of the water contact angle (WCA) to determine changes in hydrophilic performance [121]. The formation of oxygen-containing functional groups increases the surface hydrophilicity, promoting water uptake and enhancing susceptibility to hydrolytic degradation [94,113,122].

#### 2.5.3. Thermal Properties and Crystalline Structure

Methods based on thermal properties, such as differential thermal analysis (DTA), differential scanning calorimetry (DSC), and thermogravimetry (TGA), are key tools for assessing structural and compositional changes. While DSC and DTA provide information on thermal transitions, such as melting, crystallization, and glass transition, TGA monitors decomposition stages and thermal stability [39,86,123,124,125]. The importance of evaluating thermal properties and transitions relies on their close relation to the crystalline degree of a polymer, whereas assessing thermal and thermo-oxidative degradation behavior may allow for evaluating degradation kinetics. Additional techniques, such as coupling TGA with FTIR to monitor the evolution of gaseous products during degradation, are required to provide indirect insights into chemical modifications [126].

Also related to the crystalline structure, X-ray diffraction spectroscopy (XRD) is an analytical technique used to determine the degree of crystallinity in polymeric materials, quantifying the percentage of amorphous and crystalline domains in the polymer matrix and the crystallinity index [127].

#### 2.5.4. Chemical Structure

Identifying specific functional groups and tracking changes in their concentration is essential for understanding structural modifications. The appearance or disappearance of particular chemical groups offers valuable insights into reaction mechanisms and the formation of degradation products [128]. Fourier transform infrared spectroscopy (FTIR) is one of the most widely used techniques for monitoring abiotic changes at the molecular level. Because each functional group consists of different atoms and bond strengths, its vibrations are unique and can be detected and assigned accordingly to chemical modifications such as chain scission, oxidation, crosslinking, or incorporation of new functional groups that occur during degradation [86]. The two key functional groups that are usually picked to trace the degradation process correspond to carbonyl (C-O) and hydroxyl groups [66,114]. Historically, the carbonyl index (CI) has been used to assess the degree of polymer oxidation, since oxidation is considered the primary driver of polymer degradation in environments that contain oxygen [129]. However, the FTIR technique itself may not provide comprehensive details on the entire degradation process, highlighting the necessity for complementary analytical techniques such as DSC and TGA [130].

Furthermore, X-ray photoelectron spectroscopy (XPS) is a surface-sensitive technique that provides a quantitative elemental analysis, except for hydrogen and helium, and chemical state information via peak deconvolution. The tracking of surface oxidation through an increase in the O/C ratio and oxidized carbon components is a common methodology applied in oxidative nature pretreatments [131].

For the analysis of trace elements that can be leached, commonly inorganic additives, such as pigments, stabilizers, or catalytic agents, X-ray fluorescence (XRF) can be useful, as it allows non-destructive detection and quantification of these elements within the polymer matrix [132].

Additionally, methods such as nuclear magnetic resonance (NMR) can offer complementary insights into composition and molecular structure changes, such as bond cleavage of carbons in the side chains and backbone, and incorporation of oxygen molecules into the polymer. However, NMR analysis can be challenging for highly crystalline or poorly soluble polymers, making it less practical as a routine method [86].

#### 2.5.5. Molar Mass

Molar mass analysis provides essential insights into the structural integrity and degradation extent of polymers after exposure to degrading conditions. To determine these changes, chromatographic methods are useful due to their capacity to separate complex macromolecular compounds according to their interactions between the mobile and stationary phases in the chromatography column [86]. From the chromatographic techniques, gel permeation chromatography (GPC) is used to determine the number-average molar mass (*M_n_*) and weight-average molar mass (*M_w_*). Furthermore, the *M_w_*/*M_n_* ratio, also known as the polydispersity index (PDI), is a useful indicator for evaluating molar mass distribution [133,134]. It is well known that when polymers are exposed to degrading factors during their service lifetime, changes in molar mass occur [104,135]. Under degradative mechanisms, such as photodegradation, mechanical stress, or hydrolysis, polymer chains may undergo fragmentation, making GPC particularly important for determining the molar mass distribution of the resulting fragments [51]. Additionally, when GPC is not available, methods based on viscosity measurements are useful due to low experimental setup requirements. Capillary viscosimetry, in particular, is recommended because the viscosity of a polymer solution depends on the concentration and hydrodynamic size of the dissolved macromolecules. From the determination of intrinsic viscosity (*η*), the viscosity of average molar mass (*M_v_*) can be estimated [136,137].

Each of the evidence and corresponding characterization methods mentioned above sheds light on the modifications that biopolymers undergo upon exposure to degrading agents. However, to achieve a comprehensive understanding of degradation pathways, selected methods should be applied in combination [130,138], including the characterization of the pristine material and the evaluation of changes in physicochemical properties during degradation.

### 2.6. Strategies to Promote Bio-Valorization of Bioplastics

**Figure 2:**
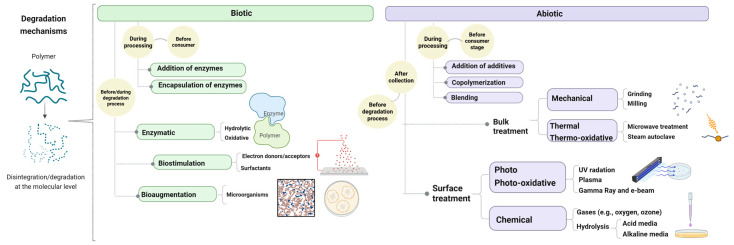
Abiotic and biotic approaches to accelerate the biodegradation of polymers: from processing modifications to treatment strategies.

Since the factors affecting bioplastic biodegradation and the evidence of biopolymer degradation were considered, various strategies have been explored in the literature to address biodegradation limitations, as shown in Figure 2. The main approaches involve modifying the biotic and abiotic degradation phases [9]. The biotic phase methods are focused on modifying the surrounding environment to activate the biodegradation of biopolymers during processing (addition or dispersion of enzymes) or during biodegradation (bioaugmentation, biostimulation, and engineering of enzymes).

Alternatively, abiotic methods involve modifying the properties of the polymer during processing or at the end-of-life stage. These modifications can include changes to surface properties such as surface energy, roughness, and hydrophilicity capacity, as well as bulk properties like crystallinity, chain mobility, and molar mass reduction.

The abiotic modifications during processing include the incorporation of small and more labile to biodegradation molecules as additives [34,35,139], such as plasticizers and compatibilizers, and the formulation of polymer blends containing more readily biodegradable components [84]. This strategy falls within the basic principles of ecodesign, which seeks to offer solutions from the earliest stages of product development [140,141,142]. For instance, PBAT exhibits a relatively low biodegradation rate [90], but degradability is enhanced when blended with more readily biodegradable polymers such as thermoplastic starch (TPS) [143,144,145]. The hydrophilic nature of TPS accelerates the biodegradation of polyester-based blends in an aerobic controlled environment under mesophilic and thermophilic conditions [146]. Also, the preparation of self-reinforced polymer composites (SRCs), consisting of both a matrix and reinforcement from the same biodegradable polymer, has emerged as a promising approach to improve biodegradability under standardized conditions (ISO 20200) [147]. However, while certain compatibilizers can improve the mechanical integrity and interfacial adhesion of biopolymer composites, they may also hinder biodegradation by limiting the accessibility of biodegradable phases to microbial or enzymatic attack [143].

On the other hand, abiotic modifications as a previous stage to biotic degradation involve a reduction in particle size, surface and bulk treatment modifications, and catalysis of chemical hydrolysis. Such alterations are very relevant, playing a crucial role in enhancing subsequent degradation performance. Principal degradation mechanisms, such as mechanical, thermal, photo-, and hydrolytic degradation, enhance the following biotic degradation by increasing the concentration of hydrophilic functional groups, reducing molar mass, or increasing the surface area available for biofilm formation [48,50,139,148].

Surface pretreatments typically alter the surface of biopolymers, increasing roughness and making some components more accessible to microbial and enzymatic activity. Photodegradation of PBAT/PLA blends enhances the colonization of *Bacillus subtilis* by increasing surface roughness and making lactic acid more accessible [149]. Moreover, technologies that promote surface oxidation lead to changes in topography and loss of functional groups, such as carbonyls, while generating carboxylic acids, diketones, and esters. These newly formed groups may enhance the adhesion of microorganisms and enzymes, thereby accelerating the degradation process [150]. However, bulk pretreatments focus on reducing overall molar mass and promoting deterioration of the whole polymer structure [9].

## 3. Abiotic Strategies and Their Integration into Bioplastic Bio-Valorization Value Chains

**Figure 3:**
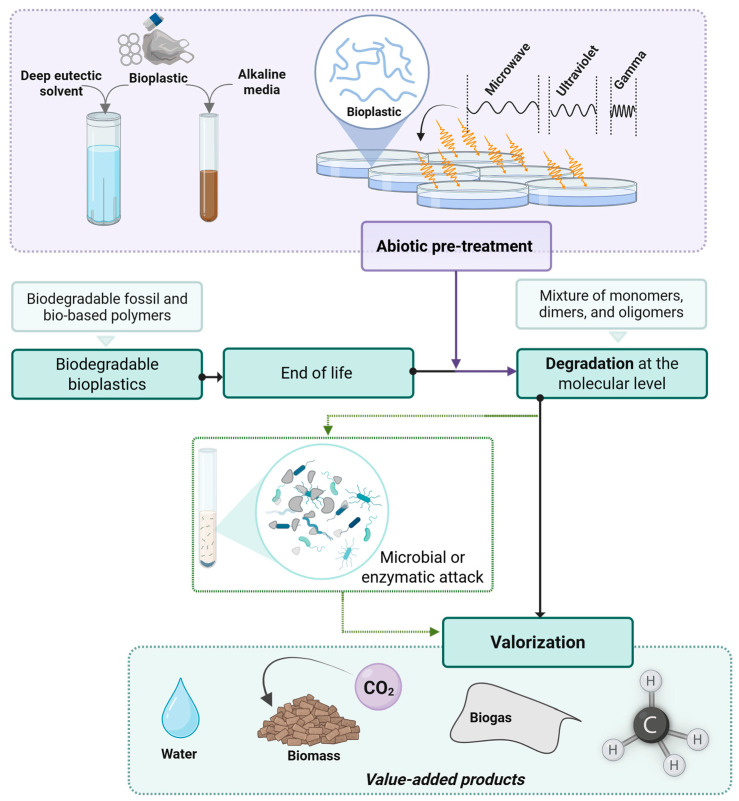
Schematic representation of abiotic pretreatments accelerating bioplastic biodegradation and value recovery at end of life.

From the perspective of accelerating ulterior biodegradation, efforts in the literature have been made to understand the consequences of abiotic treatments for biodegradable polymers, especially the enhancement of bioplastic bio-valorization opportunities [45,46,47,48,101]. Physical pretreatment methods like UV radiation, sonication, and plasma treatments have been reported to enhance microbial degradation by disrupting the macromolecular aggregate structures of bioplastics [93]. These abiotic degradation methodologies, serving as pretreatments, have emerged as effective tools to enhance the susceptibility of bioplastics to microbial attack and enzymatic degradation, as schematized in Figure 3. Considering pretreatments as processes of a physical, chemical, or thermochemical nature that are capable of enhancing the biodegradation capacity of polymers before biotic degradation [45], this section gathers different approaches described in detail, emphasizing their mode of action, advantages, and integration within circular end-of-life strategies for bioplastic bio-valorization.

### 3.1. Physical-Based Treatments

#### 3.1.1. Thermal Pretreatment

In thermal degradation, the decomposition of polymer chains is induced by exposure to elevated temperatures in the presence of oxygen (thermo-oxidative) or under limited oxidative conditions (thermal). This process is initiated by thermal energy that attacks weak points in the polymer backbone, such as structural imperfections or thermally labile bonds. In some cases, specific chemical groups such as peroxide or ether linkages can act as initiators of the degradation process, leading to fragmentation, release of oligomers, and the formation of volatile by-products [47,126,151,152].

In particular, thermal degradation occurs through the bulk of the polymer, with four distinct reactions occurring simultaneously: (1) end-chain scission or chain depolymerization reaction of C-C bonds, which involves the successive release of monomer units from the chain ends [50]; (2) random chain scission, which causes a reduction in molar mass; (3) degradation by successive reactions; (4) recombination of cyclic and linear oligomers.

Typically, thermal degradation of thermoplastic biopolymers occurs near the melting temperature (*T_m_*) [11,51]. In some cases, such as PCL, *T_m_* falls within the temperature range of mesophilic to thermophilic biodegradation processes (approximately 20–60 °C). Under these conditions, partial thermal softening may facilitate structural rearrangements that condition the polymer matrix and enhance its susceptibility to microbial attack [51].

On the other hand, for polymers such as PLA (*T_m_* ≈ 155 °C) or PHB (*T_m_* ≈ 175 °C), thermal degradation does not occur within typical biodegradation temperatures, making thermal effects negligible under standard composting or soil conditions. However, the degradation rate of PLA increases above its glass transition temperature (*T_g_* ≈ 55–62 °C), where polymer chains become more flexible and water absorption is enhanced, thereby accelerating both hydrolysis and microbial attachment [91]. Polymers with a *T_g_* below the pretreatment temperature may therefore have more labile structures, which may lead to higher biodegradation rates [9].

Complementarily, thermal treatments such as heating followed by rapid quenching have been widely employed to manipulate the microstructure of biodegradable bioplastics. This procedure inhibits the formation of ordered crystalline domains by “freezing” the polymer chains in a disordered state, thereby promoting the generation of amorphous structures. Since amorphous regions are less densely packed and more accessible to water molecules, enzymes, and other degradative agents, their presence strongly enhances the susceptibility of the material to hydrolysis and subsequent biodegradation. In addition, reduced crystallinity is often correlated with faster microbial colonization and higher rates of bio-valorization, as the amorphous phase provides more reactive sites and facilitates chain scission. Thus, the heating/quenching strategy represents a simple yet effective approach to tailor the degradability of bioplastics, aligning material design with end-of-life environmental performance. A typical process of heating and quenching was used to prepare PBSA films with lower crystallinity [92]. As a result of the thermal pretreatment, films with lower crystallinity degraded more rapidly in soil. Additionally, BA-rich segments were preferentially attacked during the degradation process, leading to a gradual decrease in BA content over time.

#### 3.1.2. Mechanical Pretreatment

When polymers are exposed to harsh environments or the action of mechanical stresses, e.g., compression, tension, and/or shear forces, mechanical degradation occurs in the form of the breakdown of polymer chains [51]. Mechanical deterioration often occurs during processing, aging, and exposure to repetitive stress during service life [11]. Mechanical degradation is particularly relevant during extrusion, milling, or blending, where powerful shearing forces, combined with temperature and pressure, can initiate structural damage with bond cleavage and formation of active radicals and consequent molar mass decrease [49,153].

**Figure 4:**
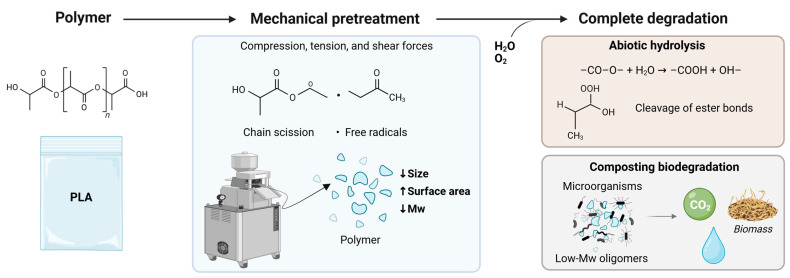
Schematic representation of the effect of mechanical pretreatment on the degradation pathway of poly(lactic) acid (PLA).

Grinding has been used as a mechanical action to alter the structure and reduce the size of biopolymers, serving as a preliminary step to improve the efficiency of subsequent degradation processes [30]. Physical scission of chemical bonds, frequently accompanied by the generation of free radicals, may take part in subsequent reactions with environmental agents such as oxygen (formation of peroxide radicals) or moisture [50]. Moreover, considering that the degradation rate of bioplastics is strongly influenced by surface accessibility and the extent of microbial colonization, the increase in surface area available to enzymatic attack may increase the kinetics of the biodegradation [11,68]. Indeed, commercial bioplastics, such as PBS/starch-based blends and PLA, exhibited limited or negligible degradation in soil without prior grinding. However, when tested in powdered form, a significant increase in degradation was observed both in short-term (28 days) and long-term (up to 2 years) soil incubation experiments [37]. As schematized in Figure 4, the combination of particle size reduction with a lower molar mass of PLA was favorable in accelerating both the biodegradation under composting and during abiotic hydrolysis for complete degradation under long-term (100 days) and thermophilic conditions (58 °C) [154]. Interestingly, particle size reduction of PLA pellets through milling and sieving to obtain powders of different particle sizes increases the biodegradation rate in the initial steps of anaerobic digestion but does not affect the ultimate methane production rate [148], unless combined with alkaline–thermal pretreatment [155].

#### 3.1.3. UV Pretreatment

UV pretreatment refers to ultraviolet radiation exposure under controlled conditions, as can be performed using UV chambers, UV lamps, or simulating natural aging. In this context, standardized methodologies such as ISO 4892:2013 set out the conditions for simulating the effects of aging that occur when plastics are in end-use environments. Under these standardized conditions, plastics are exposed to total solar radiation or filtered solar radiation using xenon arc lamps (ISO 4892-2) or fluorescent UV lamps (ISO 4892-3) [156,157].

Both photodegradation and radiation degradation refer to the breakdown of a material in the presence of O_2_ (photo-oxidative degradation) or the absence of O_2_ (photolysis), due to the exposure to wavelengths in the ultraviolet (UV), visible (Vis), infrared (IR), or gamma ranges. Radiation exposure is considered one of the main causes of structural damage in polymeric materials during service-life conditions, especially UV-Vis, and has been proven to be effective in altering the structural stability of polymers [11]. The control parameters to be monitored in a given UV pretreatment include wavelength range, irradiance level of the selected irradiation source, exposure time, temperature, and presence of oxygen and humidity in the medium.

In general, photodegradation involves the absorption of UV energy by a polymer backbone, resulting in the disruption of its structural integrity through processes such as photoionization, oxidative reactions, and chain scission [158]. This phenomenon typically proceeds via well-established Norrish Type I (photo-ionization) and Norrish Type II (polymer chain scission) reactions [50,159,160], which involve the generation of reactive free radicals and ultimately lead to the fragmentation and breakdown of the polymer chains [51]. Photodegradation can also lead to crosslinking reactions [90], limiting further biodegradation.

**Figure 5:**
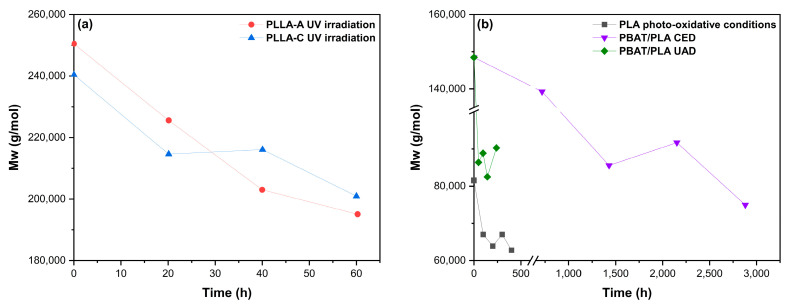
Representative variations in the molar mass of different polymers during irradiation and degradation treatments. Data correspond to (**a**) amorphous PLA films exposed to accelerated UV ageing and (**b**) PLA/CaSO_4_ composites under photo-oxidative conditions, PBAT/PLA mulch films subjected to UV-accelerated degradation (UAD) and cultivation environment degradation (CED). Lines are given for a visual aid. Data sourced from [98,161,162].

Accelerated UV exposure studies have demonstrated that photodegradation can promote bulk erosion and molar mass reduction in biopolyesters, as shown in Figure 5. Tsuji et al. (2006) showed that under controlled UV irradiation (255 W·m^−2^, 45 °C, 65% RH) both PLA and PCL films underwent chain scission despite differences in crystallinity, with PCL showing higher photodegradability and reductions in elongation at break [102]. Similarly, Xie et al. (2022) demonstrated that indoor accelerated UV aging (340 nm, 1.1 W·m^−2^, 60 °C) effectively simulates outdoor field degradation of PBAT/PLA mulch films, confirming that UV exposure is a key factor driving molecular breakdown in these blends [98]. However, PBAT presence in PBAT/PLA moderates the photodegradation rate of PLA under UV-C (254 nm), although significant mechanical deterioration and molar mass reduction still occur with prolonged exposure [150]. Indeed, photodegradation of PLA depends on the components present in the blends, and systems containing higher TPS proportions exhibit the most pronounced loss of mechanical integrity under UV aging, reflecting the greater photochemical susceptibility of the starch-rich phase in PLA/TPS blends [117].

In the photodegradation of PLA, the Norrish Type I mechanism involves the breakdown of the carbonyl group, dissociating into free radicals and chain cleavage fragments, resulting in a decrease in molar mass [163,164]. The Norrish Type II mechanism, where a photon is absorbed by the polymer chain, causes a C-O bond cleavage and the subsequent formation of hydroperoxides that lead to the degradation of PLA into corresponding carboxylic acids and esters comprising double C=C bonds [52]. Both mechanisms have been observed when the degradation took place at 254 nm [165], due to there being enough energy capable of breaking the chemical bonds in PLA. At wavelengths in the UV-C range, radiation possesses sufficient energy to induce the disintegration of PLA [166] and bulk deterioration of PBAT-based biopolymers [100] when bioplastics are directly exposed to such wavelengths.

Under natural conditions where wavelengths typically exceed 300 nm, photodegradation via a Norrish Type II mechanism is unlikely to occur, as the energy provided is insufficient to cleave the polymer backbone of aliphatic biopolymers such as PLA, resulting in limited degradation [52,162]. Although PLA itself does not absorb radiation above 300 nm, the presence of chromophore impurities or additives can absorb UV light in this range and generate reactive radicals that initiate degradation processes in the polymer matrix [167]. However, the use of some additives, such as MMT (montmorillonite), interferes with the photodegradation process, delaying the oxidation, even at 254 nm wavelengths [52].

In product development, particularly in medical applications, the textile industry, and biodegradable packaging, it is essential to understand how UV radiation influences the physical and chemical properties of materials, as this directly affects their performance and lifespan. Particularly in medical applications, it is relevant whether biopolymers in contact with human tissues degrade and whether the possible degradation products could have adverse effects on the human body. In medical and pharmaceutical applications, polylactide materials are sanitized with UV radiation at a 254 nm wavelength, accelerating the degradative process [52]. Additionally, UV radiation has been studied to elucidate material deterioration in outdoor applications like agriculture to assess the environmental impact of degradation compounds and the service life of bioplastics under aging conditions [98,149].

Altogether, several studies have demonstrated that integrating UV-based pretreatments with biological valorization techniques can significantly accelerate the degradation of bioplastics in both aerobic and anaerobic environments. Photodegradation of PBAT/PLA blends has been shown to increase surface roughness and expose more readily accessible chemical groups, such as lactic acid. These surface modifications enhance the colonization of specific microorganisms, particularly Bacillus subtilis. Consequently, the combination of UV-induced photodegradation and targeted microbial activity results in accelerated polymer fragmentation and mineralization [149]. Combination strategies of UV irradiation with high-frequency ultrasonication enhanced PLA biodegradability under mild conditions [168]. However, prolonged photodegradation exposure may lead to crosslinking that limits the accessibility of polymer chains to microbial attack [90]. Table 5 summarizes selected studies that illustrate the synergistic effects of UV pretreatments combined with biological degradation processes. These approaches take advantage of UV-induced structural modifications to enhance microbial colonization and enzymatic accessibility.

#### 3.1.4. Ionizing Radiation Pretreatments

Two well-known types of ionizing radiation that are frequently employed in the food, pharmaceutical, and medical industries for material treatment, sterilization, and polymer modification are gamma (γ-rays) and electron beam (e-beam) irradiation. Both methods use high-energy radiation that can change a material’s chemical structure by producing reactive species such as oxidative molecular compounds, free radicals, or hydrated electrons [171].

High-energy photons, usually released by isotopes such as ^60^Co or ^137^Cs, are used in gamma irradiation. In a nuclear reactor, ^59^Co absorbs neutrons to create radioactive ^60^Co, which decays and releases gamma rays. With energy ranging from 1.17 to 1.33 MeV, these gamma rays are extremely penetrating electromagnetic waves that can infiltrate materials up to tens of centimeters thick. Gamma irradiation is therefore especially well-suited for treating big, heavy, or pre-packaged materials, particularly for cold sterilization, because of its reliability and uniformity. Depending on the product’s size, density, and intended dosage, it can constantly apply radiation from minutes to several hours. Nevertheless, it may cause molecular-level changes in polymers, such as crosslinking or degradation [172,173,174].

In contrast, thermionic emission from an electron gun generates high-speed electrons through an electron beam (e-beam) irradiation. In a waveguide system, microwave radiofrequency energy is used to accelerate these electrons. The electrons receive energy from the electromagnetic field and are propelled into a drift tube, where they accelerate to high speeds. E-beams, as opposed to gamma rays, emit radiation in short, pulsed bursts, which enables extremely quick dosage distribution, usually in a matter of seconds. They are perfect for surface-level or smaller-sized products, though, because of their more restricted penetration depth, which usually only reaches up to 15 cm. E-beam irradiation is a very effective, clean, and simple method of irradiation [175,176].

Both gamma and e-beam irradiation techniques operate by breaking molecular bonds and producing reactive species, which start radiochemical reactions in polymers, including scission, crosslinking, and radiolysis. E-beam irradiation provides higher precision and quicker processing times, but it has less depth than gamma irradiation, which is superior in terms of homogeneous dose distribution and deep penetration. As a result, the material properties, size, and processing are factors determining which option is more appropriate [171]. As summarized in Table 6, gamma irradiation and electron beam exposure have been widely investigated primarily for polysaccharides and polyesters.

Gamma irradiation doses are typically ranging from 20 to 50 kGy, leading to modifications of chemical structure detectable by FTIR, like shifts of -OH and C=O bands and reduced crystallinity [173,177]. Nevertheless, at higher doses of irradiation, chain scission may occur. In poly(vinyl alcohol)/bacterial cellulose (PVA/BC) films, transparency was maintained; however, increased yellowness and redness were noticed, indicating chromophore formation. In that composition, degradation of bacterial cellulose was evident and proved by the splitting of the 1081 cm^−1^ band, even though mechanical and barrier properties remained intact up to 50 kGy [173]. Similar behavior was confirmed in poly(3-hydroxybutyrate)/poly(ethylene glycol) (PHB/PEG) blends, in which, at low levels of degradation, mechanical properties (tensile strength and elongation at break) were improved, whereas over 40 kGy, significant degradation was reported simultaneously with reduced water vapor permeability [177].

E-beam irradiation has also been effectively applied, for example, for pectin/polyacrylamide (PEC/PAAm) and fish gelatin/glycerol films. Doses in the range from 5 to 30 kGy resulted in crosslinking of polymer chains, decreased crystallinity, and increased hydrophilicity of the films’ surfaces. A medium irradiation dose of 30 kGy led to the optimal properties of the films (optimal mechanical performance and stability), enhancing the shelf-life of the material [178]. In gelatin/glycerol-based films, an irradiation dose above 60 kGy induced free radical formation and crosslinking in the amorphous phase without affecting crystallinity [179].

For poly(lactic acid) (PLA) films, both gamma and E-beam irradiation pretreatment led to chain scission and reduced crystallinity, as well as visible yellowing/greening of the film and weakened mechanical performance, especially relevant for gamma irradiation doses above 45 kGy. In this regard, controlled irradiation conditions enhanced mineralization in a simulated aerobic composting environment, suggesting their potential for being used in biodegradable packaging applications as an end-of-life scenario [180,181].

Overall, ionizing radiation may promote crosslinking of polymer chains or chain degradation, depending on the polymer type and applied doses. Moderate irradiation doses, typically up to 30 kGy, can enhance mechanical performance and water resistance and consequently the shelf-life of the material, while exposure to higher doses promotes oxidative degradation and loss of crystallinity, which may contribute to further biodegradation processes in bioplastics.

#### 3.1.5. Plasma Treatment

Plasma treatment has been reported as an effective technique for generating reactive oxygen species (ROS) and inducing surface modifications in plastic materials with relatively short-term performance [93]. Depending on the energy and temperature of the charged particles involved, plasma systems are typically classified into two main types: thermal (equilibrium) plasma and non-thermal (non-equilibrium) plasma [182]. Among them, non-thermal plasma (NTP) has gained considerable attention for its ability to modify surface chemistry without significantly increasing the overall temperature. NTP occupies a substantial volume within the discharge medium and operates near ambient conditions, enabling the generation of reactive species in the material’s surface.

Principally, several types of non-thermal plasma have been explored for polymer surface modification, including radio frequency (RF) plasma, corona discharge, microwave plasma, direct current (DC) glow discharge, atmospheric-pressure plasma jets, and dielectric barrier discharge (DBD) plasma systems, with the corona and DBD being the most commonly used discharge methods [183]. The main distinction lies in the mechanism and medium of discharge generation. For instance, corona discharge and DBD plasmas operate at atmospheric pressure with dielectric barriers that limit current and stabilize the plasma, whereas RF and DC glow discharges require vacuum conditions and electrode configurations that influence the discharge behavior and energy transfer.

Corona discharge is distinguished by the generation of plasma in areas of high curvature in the electrodes due to the strong potential gradient. Electrons from the electron avalanche and positive ions recombine to form neutral atoms, giving the corona discharge its glow. DBD is formed between two electrodes, and at least one of them is covered with a dielectric layer. The dielectric material serves to limit the charge transported during the discharge and ensures a uniform distribution over the electrode surface [184]. Through the high-voltage electrical discharge, the exposed air at the surface of the material is ionized, and when a polymeric material is placed in the discharge path, the electrons generated in the discharge impact the plastic surface with energies two or three times higher than necessary to break the chemical bonds on the surface of most substrates [185].

As a result of air ionization, free radicals such as reactive oxygen species (ROS) and reactive nitrogen species (RNS), including ozone (O_3_), hydroxyl radicals (·OH), peroxides (H_2_O_2_), and nitrogen–oxygen radicals, are generated. These substances oxidize the substrate surface to form various chemical functional groups, such as carbonyl and hydroxyl groups, which increase the hydrophilic character of polymers [186,187].

Generally, plasma treatment is a very convenient strategy to incorporate functional groups in a polymer surface with a limited effect on bulk properties [131,188]. Surface modifications and localized thermal effects generated in plasma discharge have a positive effect on the degradation of organic compounds [189]. Through NTP treatment, the biodegradation of traditional polymers such as low-density polyethylene (LDPE) increased due to an increase in oxidative species, causing better cell adhesion and acceptance on the polymer sample surface [190]. Also, treatments using corona discharge and RF configurations have demonstrated effectiveness in introducing polar functional groups onto the surface of PLA, thereby enhancing its wettability and facilitating subsequent hydrolytic degradation under accelerated weathering conditions [191].

Table 7 summarizes selected plasma treatment strategies for enhancing the biodegradation of biopolymers. As a strategy for the end of life of bio-based products, DBD-NTP has been proven to slightly affect physico-chemical properties of high-rigidity biodegradable plastics, such as PLA-based cutlery items [192], with complete disintegration after 20 days of high-voltage exposure and composting in a home-scale reactor. Air-short plasma treatment enhanced the surface properties by incorporating polar functional groups and increasing surface roughness. These surface modifications have been associated with improved degradation performance, facilitating both subsequent hydrolytic degradation [188,191] or microbial activity [192,193].

### 3.2. Chemical-Based Pretreatments

Chemical pretreatments involve the use of solutions with differential chemical activity (acidic, basic, or oxidizing agents) capable of altering the structure of the polymer through mechanisms such as hydrolysis, oxidation, or even solvolysis-driven depolymerization. The main control parameters in chemical and hydrolytic pretreatments include temperature, exposure time, pH level, and the agitation mechanism. These variables critically influence the rate and extent of polymer degradation by modulating the diffusion of reactive species and the accessibility of polymer chains to hydrolytic or oxidative attack. In this context, surface or bulk erosion depends deeply on the diffusivity of the solution inside the polymer matrix; the degradation rate of labile functional groups; and the dimensions, principally the thickness, of the specimen [194].

#### 3.2.1. Hydrolytic Approaches

**Figure 6:**
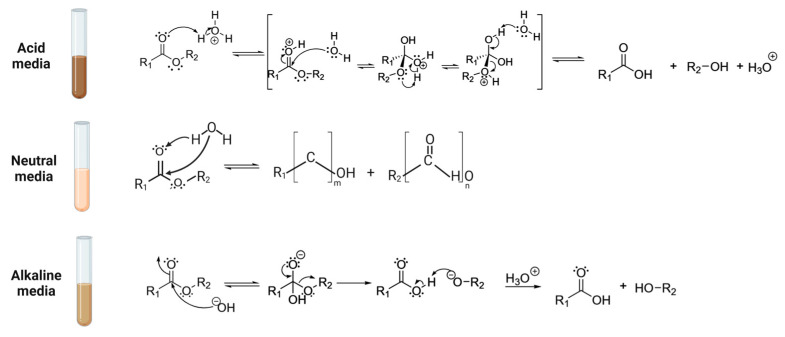
Mechanisms of hydrolytic degradation of polyesters under acidic, basic, and neutral conditions.

Hydrolytic degradation may occur during the exposure of a polymer to an aqueous medium containing water molecules, as schematized in Figure 6. This process consists of breaking labile chemical bonds, such as ester, amide, or anhydrous bonds, by reacting with water, leading to a reduction in the molar mass and to the generation of oligomers or monomers [16,41,53]. Ester bonds in biopolymers, such as aliphatic polyesters derived from poly(hydroxy-acid)-type homopolymers and copolymers, degrade in the presence of water molecules [113], leading to chain scission and polymer fragmentation [147]. During the hydrolysis of biopolymers, the degradation products induce significant changes in the pH of the degradation medium, directly affecting the kinetics of the reaction [195]. Depending on the balance between water diffusion and the hydrolysis rate, this process may occur either at the surface or through the bulk of the material, with bulk erosion prevailing when water diffusion is faster than the hydrolytic reaction [53,194,196,197].

For PLA, a high rate of degradation is achieved when the temperature is around the glass transition of the polymer, i.e., 58 °C [147,198]. Nevertheless, prolonged hydrothermal exposure at higher temperatures (e.g., 85 °C) can lead to chain cleavage in the amorphous regions, rearrangement, and recrystallization, increasing the crystalline fraction [199,200].

Alkaline aqueous conditions have been widely studied in the context of abiotic degradation, particularly for aliphatic polyesters such as PLA-, PHB-, and PBAT-based blends [111,201], even causing complete disintegration in short-term conditions [99]. In such systems, hydroxide ions (·OH) catalyze the cleavage of ester bonds, accelerating the hydrolytic depolymerization of the polymer chains into low-molar mass fragments. This molecular breakdown significantly enhances the susceptibility of the polymer to subsequent biodeterioration under biological valorization conditions [148].

As is well known, the temperature of the degradative media plays a critical role in abiotic treatment. In the case of PBAT-based materials, alkaline pretreatment under thermophilic conditions has been shown to enhance subsequent anaerobic digestion. Specifically, treatment with a solution of NaOH at 70 °C for 48 h increases the initial methanogenic activity, which is attributed to a reduction in molar mass and the formation of porous structures [201]. In line with this, an efficient pretreatment for PLA pellets consists of heating at 70 °C for 48 h in the presence of an alkaline aqueous medium of 2.5% *w*/*v* Ca(OH)_2_, which leads to a very high subsequent methane potential and a biodegradation yield of 73% in 30 days [148]. However, thermo-alkaline pretreatment with NaOH under mesophilic conditions did not enhance PLA and PBAT pellet conversion into CH_4_ or CO_2_ [201]. This highlights that the effectiveness of a chemical pretreatment is an interplay between temperature, chemical activity, treatment time, and specimen size and morphology [131].

#### 3.2.2. Non-Oxidative Solvolytic Strategies

Pretreatment with non-oxidizing chemicals has emerged as an effective strategy for improving the biodegradability of plastic waste by modifying properties such as morphology, crystallinity, and molar mass. Deep eutectic solvents (DESs) have attracted significant attention owing to their low toxicity, high biodegradability, and chemical versatility. These solvents, formed by a hydrogen bond donor and acceptor, offer a sustainable and tunable alternative to traditional solvents for the pretreatment of both fossil- and bio-based plastics [202].

DES-based pretreatments have been shown to enhance surface hydrophilicity and promote microbial colonization. For instance, a combined approach of bioaugmentation and the dip-coating method with a DES composed of choline chloride and glycerol (ChCl:Gly) enhances wettability and biofilm formation on PET and PLA (from bottles, films, and cups). Improvement in degradation in terms of mass loss was found under both aqueous (e.g., PLA film ~36% vs. 21% of pristine material) and lab-scale composting conditions (e.g., PLA film ~64% mass loss at 42 d), while an increase in the cumulative CO_2_ was observed. Although the authors did not report the polymer mineralization percentage, the increased CO_2_ evolution, together with mass loss, supports that DES pretreatment enhances biodeterioration and bioassimilation [120].

Synergistic strategies have also demonstrated promising results, where the integration of DES (ChCl/lactic acid) pretreatment with UV-C irradiation and biosurfactants significantly enhances PLA mineralization, resulting in increased CO_2_ evolution and accelerated degradation kinetics [203]. Microwave-assisted ternary DES (ChCl/Gly/urea) pretreatment improves PET enzymatic hydrolysis, achieving monomer recovery rates of up to 16% [204]. These effects are attributed to the ability of the DES to interact with ester bonds via hydrogen bonding, which lowers the activation energy required for cleavage. In some formulations, the inclusion of metal ions provides Lewis acid catalysis to accelerate degradation [204].

In addition to DESs, other non-oxidizing chemical pretreatments have been proven to be effective. Pretreatment of PET with anionic surfactants increases enzyme accessibility and results in a 120-fold improvement in PETase activity [205]. In another strategy, melt-blending low-density polyethylene (LDPE) with fatty acids softens the polymer matrix, enabling fungal colonization and oxidative degradation, which leads to a fourfold increase in substrate oxidation [45]. These studies show promise for applying analogous strategies to biodegradable bioplastics and enhancing bio-valorization performance. A comparative overview of the mentioned strategies is provided in Table 8, which summarizes the key details. This highlights the versatility of emerging non-oxidizing pretreatments for polymer biodegradation.

#### 3.2.3. Oxidative Pretreatment

Beyond the hydrolytic and non-solvolytic pretreatments, oxidative strategies promote the formation of oxygen-containing functional groups and chain scission through the generation of reactive oxygen species (ROS). Among them, ozone-based oxidation and advanced oxidation processes (AOPs) represent two complementary approaches, differing in the mechanism and complexity of oxidant generation.

Ozonation relies on the high reactivity of ozone (O_3_) to induce modifications and even degradation in polymers. Due to its high redox potential (2.07 V) and ability to act as both a nucleophile and electrophile through resonance stabilization [206,207], ozone interacts with polymers via two main routes: (i) direct attack of molecular ozone, involving oxidation/reduction, electrophilic substitution, nucleophilic substitution, or cycloaddition reactions, and (ii) indirect oxidation mediated by hydroxyl radicals (·OH) generated during ozone decomposition. These processes typically follow initiation, propagation, and termination stages [207]. Generally, ozonation induces both polymer chain scission and crosslinking, accompanied by an increase in oxygen-containing functional groups on the surface that increases the hydrophilicity of the polymer.

The influence of ozone treatment on the polymer surface and performance is strongly related to the time of ozonation and the type of polymer. A short time of ozone exposure is called ozone sterilization rather than ozone degradation [208]. However, prolonged treatments have been reported to cause bioplastic degradation.

For PLA/LDPE blends, ozonation (8–16 h) increased crystallinity while reducing both melting and glass transition temperatures [209]. Although mechanical properties were not directly measured, the authors suggested possible embrittlement due to amorphous phase disruption. In PLA fibers, short exposures (<10 min) improved whiteness (6% increase), water absorbency (20% reduction in wetting time), and flexibility (16% reduction in flexural rigidity), with negligible loss of burst strength [210]. Prolonged treatment (60 min) led to an ~10% strength reduction, though surface electron micrographs showed no significant morphological damage.

Ozonation of PHB for long-time exposure has been reported to significantly alter its structural and mechanical behavior [211]. However, instead of enhancing biodegradability as initially hypothesized, ozone exposure promoted the accumulation of oxygen-containing groups and interchain crosslinking [212]. Calorimetric analyses revealed that ozonation preferentially affects amorphous domains, leaving crystalline macromolecules unaltered. The increase in the degree of crystallinity and the changes in melting temperature, related to the reorganization of the amorphous phase and the scission of the most stressed sections of the polymer chains after ozone exposure, lead to a more ordered arrangement of macromolecules in the crystalline regions, hindering the material’s biodegradability [213].

Cassava starch exposed to ozonation yielded modified cassava starch (MCS) with lower crystallinity, increased carboxyl functionality, and improved elasticity [214,215]. In blends with PVA/NR containing 20% MCS, FTIR confirmed enhanced hydroxyl and carboxyl groups, while XRD and IR indices demonstrated reduced crystalline order. Biodegradation assays showed faster degradation for samples with >15% MCS, which fully decomposed within 30 days [216]. Therefore, ozonation accelerated the biodegradation of cassava starch due to its lower crystallinity and the presence of additional carboxyl groups, which improved its hydrophilicity.

As discussed above, the effectiveness of ozonation strongly depends on the type of material and the treatment conditions. Nevertheless, only a limited number of studies have addressed the effect of ozone treatment on material biodegradability, highlighting the need for further investigation (Table 9). As a result of environmental degradation driven by natural or artificial factors, plastics undergo progressive fragmentation, producing smaller particles that persist in environmental media and affect ecosystems due to their long-term durability. Definitions currently accepted in the scientific community define microplastics (MPs) as synthetic organic polymers that are not naturally derived, with particle sizes of 0.001–5 mm, and nanoplastics (NPs) as plastic particles at the nanometer scale (<0.001 mm) [217,218]. For MPs’ environmental remediation, several methodologies, including chemical, physical, and biological treatments, have been proposed. However, these strategies often show low removal efficiencies and rarely achieve complete mineralization, focusing instead on physical separation or chemical aggregation rather than chemical degradation [219].

Additionally, advanced oxidation process (AOPs) are radical-driven pretreatments that involve the generation in situ of highly reactive oxygen species, such as hydroxyl (·OH) or sulfate (SO_4_^−^·) radicals, through activation sources. The radicals generated can catalyze the breaking down of resistant chemical bonds. Classifications reported in the literature are made according to the source of production radicals: (a) the UV–hydrogen peroxide process, (b) Fenton and photo-Fenton processes, (c) ozone-based processes, (d) photocatalysis, and (e) sonolysis [219,220,221].

AOPs have traditionally been successfully applied in wastewater treatment [222] and environmental decontamination of recalcitrant compounds, including persistent microplastic pollutants [207,223,224]. The highly reactive species provoke chain scission, decreasing molar mass, and also lead to the fragmentation of microplastics into smaller organic molecules [225]. However, the mineralization into water (H_2_O) and carbon dioxide (CO_2_) of commodities’ MPs requires high-energy consumption technologies, such as a combination of hydrothermal and Fenton setting [226]. Furthermore, the generation of micro-sized fragments is not confined to plastic commodities but also occurs in bioplastics, while incomplete degradation can result in the generation of biodegradable microplastics (BMPs) [227].

Although originally developed for aqueous systems, the underlying oxidative principles of AOPs can be extended to the biodegradation of BMPs. A combined pretreatment system of ground and sieved PLA/PBAT-based bags (particle size < 2 mm) with a heterogeneous Fenton-like system of magnetite (Fe_3_O_4_) and hydrogen peroxide (H_2_O_2_) was applied in situ at the start of composting. Surface reactive oxygen species (ROS) promoted oxidative aging of the polymer, inducing surface carbonyl formation, embrittlement, and random chain scission. Even though no mineralization percentage was reported, the increase in cumulative CO_2_ from the compost pile (10–14%) suggests enhanced biodegradation activity [228]. However, while these results are promising, more evidence on the specific increase in mineralization and the correct management of BMPs, as well as the control of fragmentation and the scalability of AOPs as pretreatments, is required.

### 3.3. Monitoring Degradation and Technical Implications for Abiotic Pretreatments

As highlighted above, evaluating the physico-chemical modifications induced by abiotic pretreatments is essential to understand their contribution to subsequent biodegradation. In this context, establishing clear and comparable evaluation criteria is of high relevance. Based on a literature review, Table 10 gathers the most informative physico-chemical indicators used in the literature to track abiotic pretreatment effects on bioplastics, together with the primary characterization techniques applied to detect them. An “×” highlights cases where independent studies report informative and reproducible signals for a given pretreatment, helping readers prioritize which metrics to measure.

Physical abiotic pretreatments alter the structure of bioplastics to enhance subsequent biodegradation, but their technical viability differs depending on the physical input applied. Mechanical methods such as milling or grinding increase surface area, yet they demand considerable energy input and may generate heterogeneous particle sizes that hinder reproducibility and process control. Thermal pretreatments can accelerate ester hydrolysis and reduce molar mass, but their implementation is limited by long exposure times. Irradiation-based methods (UV, gamma, and electron beam irradiation) provoke chain scission; however, competing crosslinking reactions in semicrystalline biopolymers demand careful dose control. Gamma irradiation requires specialized facilities, whereas UV systems demand periodic lamp replacement and significant energy input for long exposures, and e-beam treatment, although faster, offers limited penetration depth. Altogether, most evidence remains at the laboratory scale, with uncertain performance under real waste management conditions.

Chemical pretreatments of aliphatic biopolyesters such as PLA, PBAT, PBS, and PHB require the contribution of temperature to be effective, as their performance depends on chain mobility and on the accessibility of ester groups located in the amorphous regions. Temperatures near or slightly above the glass transition facilitate segmental motion and promote the diffusion of water, ions, or reactive species, thereby accelerating hydrolytic and solvolytic pathways. These mild temperatures, typically below 80 °C, do not entail a high energy demand; however, they often require long exposure times, up to 48 h in the case of PLA, to produce significant improvements in subsequent biodegradation. Moreover, although alkaline or DES-based pretreatments have proven effective, their practical implementation remains limited due to the cost of reagents.

On the whole, it is technically viable to induce degradation before biological valorization, as has been proven at the lab scale, though there is a low Technology Readiness Level for some biopolymers. The limits of current standards, the difficulty of reproducing industrial conditions in a laboratory environment, and the need to scale coupled pretreatments remain major barriers, along with uncertain performance under real waste management conditions. These constraints underscore the importance of developing and validating abiotic pretreatments capable of reliably improving bioplastic degradation beyond the laboratory scale.

Indeed, there is a niche for further research to ensure cradle-to-cradle biocircular economy pathways for more biopolymers, as well as room for development at the pilot scale of combinations of abiotic pretreatments within waste management facilities, as promising strategies to enhance bioplastic degradation. This approach would require specific life cycle analysis and life cycle cost estimates [229] in order to prioritize further scale-ups, which is beyond the scope of this review, inviting further research.

**Table 10:** Relevant degradation indicators and corresponding techniques for abiotic degradation of bioplastics. Arrows (↑/↓) represent an increasing or decreasing performance for the selected indicator. Crosses (×) are used to highlight cases where independent studies report informative and reproducible signals for a given pretreatment.

Evidence of Degradation	Key Indicators	Pretreatments Technologies							Ref.
		Physical				Chemical			
		Thermal	Mechanical	UV	Plasma	Hydrolytic	Non- Oxidative	Oxidative	
**Macroscopic appearance**									
Gravimetry	↓ Mass loss		×	×		×	×	×	[99,196]
Color variation	b* parameter variation			×				×	[100,115]
**Microscopic morphology and surface properties**									
Scanning electron microscopy (SEM)	Structural defects	×	×	×	×	×	×	×	[148,192]
Polarized optical microscopy (POM)	Morphology of spherulite variation			×					[102]
Water contact angle (WCA)	Hydrophilic performance variation			×	×	×		×	[131,192]
**Thermal properties and crystalline structure**									
Tensile tests (stress, elongation to break)	↓ σ_ty_	×	×	×		×			[102]
X-ray diffraction (XRD)	Mean size of crystalline domains	×		×		×		×	[199]
Differential scanning calorimetry (DSC)	T_m,_ T_c,_ T_g,_ and X_cr_ variation	×	×	×		×		×	[99,209]
Thermogravimetric analysis (TGA)	↓ T_dg_ ↑ % residue	×	×			×			[200]
**Chemical structure**									
Fourier transform infrared spectroscopy (FTIR)	Variation in carbonyl groups	×	×	×		×	×	×	[120,149,155]
Nuclear magnetic resonance (^1^H NMR)	Soluble oligomers presence					×			[201,230]
X-ray photoelectron spectroscopy (XPS)	Oxygen-containing groups (↑ O/C)			×	×			×	[131,193]
**Molar mass**									
Gel permeation chromatography (GPC)	↓ *M_n_*, *M_w_*	×	×	×	×	×		×	[149,164]
Capillary viscosimetry	↓ IV					×			[137]

## 4. Conclusions

The limitations in bioplastics’ bio-valorization highlight the need for complementary strategies that modulate the ability to degrade these materials under particular end-of-life management scenarios. In this context, several methodologies for enhancing the biodegradation of bioplastics have been reviewed from an abiotic perspective, so-called abiotic pretreatments.

The abiotic pretreatments based on physical approaches have proven to be effective in inducing structural modifications in bioplastics, which lead to reductions in molar mass, mechanical deterioration, and increased surface roughness. Although these changes are confined to surface levels, they are sufficient to enhance microbial colonization and enzymatic accessibility, thereby accelerating subsequent biodegradation in both aerobic and anaerobic environments. Compared with UV, gamma rays and e-beams provide a more uniform and deeper dose distribution, which translates to effective treatment of bulk materials. Ozonation and non-thermal plasma are surface pretreatments that modify polymer chemistry by introducing oxygen-containing groups and increasing hydrophilicity. Their impact on biodegradation depends on the polymer’s structure, amorphous regions being more labile to pretreatment and generally favoring faster biodegradation. Nevertheless, more investigation is required to explicitly connect physical-based pretreatments that improve the mineralization rates of bioplastics.

Chemical pretreatments, notably hydrolytic pretreatment in alkaline media, have been shown to alter the structural stability of bioplastics and to enhance subsequent biodegradation during thermophilic anaerobic digestion. Abiotic pretreatment modifications, such as a reduction in molar mass and an increase in porosity, increase initial methanogenic activity in some biopolyesters. Effectiveness depends on the coupling of temperature, pH, exposure time, and specimen thickness/morphology. Even though non-oxidizing and oxidative strategies also induce abiotic modifications, studies rarely report mineralization metrics that directly link those modifications to higher biodegradation rates. Overall, alkaline hydrolysis currently offers the most consistent, directly evidenced bridge from abiotic modification to enhanced mineralization under thermophilic anaerobic conditions.

Future works should prioritize pilot- and industrial-scale validation, with a focus on energy efficiency, operational costs, and environmental sustainability, to consolidate the practical applicability of these pretreatment strategies in real waste management systems.

## Figures and Tables

**Table 1:** Thermo-mechanical properties of common biodegradable bioplastics.

Bioplastic	Feedstock Nature	Monomer Structure	*T_g_* (°C)	*T_m_* (°C)	*TS* (MPa)	*E* (MPa)	*ε_B_* (%)	Refs.
PHA’s	Bio-based	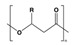	−30 to 10	70 to 170	18 to 24	700 to 1800	3 to 25	[15,16]
PBS	Bio-based	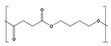	−40 to −30	115	35	300 to 500	560 to 800	[17,18]
PLA	Bio-based	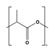	40 to 70	130 to 180	48 to 53	3500	30 to 240	[15]
PBAT	Fossil-based	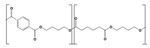	−30	110 to 115	34 to 40	2300 to 3300	1.2 to 2.5	[15]
PCL	Fossil-based	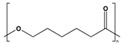	−60	59 to 64	4 to 28	390 to 470	700 to 1000	[15,16]

Glass transition temperature (*T_g_*), melting temperature (*T_m_*), tensile strength (*TS*), tensile modulus (*E*), and elongation to break (***ε_B_***).

**Table 2:** Overview of recent reviews on plastic degradation. Arrows (↑, ↑↑ and ↑↑↑) represent a qualitative scoring system to indicate how extensively each topic has been covered.

Ref.	Reviewed Polymer	Factors Affecting Degradation	Abiotic Pretreatments	Biotic Pretreatments	Degradation Mechanisms	Degradation Indicators	Pretreatment– Valorization Integration
[44]	HDPE, LDPE, PP, PET, PS, PU, PVC	↑↑↑		↑	↑↑↑	↑	
[47]	HDPE, LDPE, PP, PS, PET, PBS	↑	↑↑	↑↑	↑	↑↑↑	
[48]	LDPE, PET, PS, PP, PVC	↑	↑	↑↑↑	↑↑		↑
[46]	PE, PP, PS, PBS, PLA, Starch blends	↑↑↑	↑↑↑	↑↑↑	↑↑	↑↑	↑
[45]	PET, LDPE, HDPE, PP, PC, PUR, PVC, PLA	↑↑	↑↑↑		↑	↑↑	↑↑
[41]	PLA, PBS	↑↑↑	↑	↑	↑↑↑	↑↑	
[9]	PLA, PBAT, PCL, PBS, PHB	↑	↑↑↑	↑↑↑	↑	↑↑	↑↑

**Table 3:** Key differences between aerobic and anaerobic degradation mechanisms.

Characteristics	Aerobic Biodegradation	Anaerobic Biodegradation
Electron acceptor	Oxygen	Nitrates, sulfates, or the organic compounds themselves
Microorganisms	Aerobic bacteria, fungi	Anaerobic bacteria, archaea
Process efficiency and rate	Typically faster due to the high redox potential of oxygen; efficient breakdown of polymers	Slower; limited by the availability of electron acceptors and slower metabolic rates
Final degradation products	CO_2_, H_2_O, and simpler organic acids	CH_4_ (in methanogenesis), H_2_S, and other reduced products
Environmental implications	Lower GHG potential; widely used in composting systems	Higher GHG emissions due to methane, applicable to biogas production and anaerobic digesters
Common applications	Composting, aerobic soil biodegradation	Anaerobic digestion of organic waste, landfill environments

**Table 4:** Pass criteria and environmental conditions for composting and anaerobic valorization at laboratory scale.

Standard	Tested Environment	Measured Outcome	Conditions	Pass Criteria/ Requirements	Ref.
ISO 14855	Industrial composting	Mineralization	T: 58 ± 2 °C Oxygen: aerobic RH: 50% CO_2_ flow rate: 10 mL/min Compost inoculum from municipal solid waste	>90% CO_2_ vs. microcrystalline cellulose Duration: 180 days Reached plateau	[73]
	Home composting	Mineralization	T: 28 °C Oxygen: aerobic Compost inoculum from municipal solid waste	>90% CO_2_ vs. microcrystalline cellulose Duration: 1 year/365 days Reached plateau	
ISO 20200	Industrial composting	Disintegration	T: 58 ± 2 °C Oxygen: aerobic Compost inoculum from a municipal or industrial aerobic composting plant	90% of the initial mass passes through a 2 mm sieve Duration: from 45 to 84 days	[74]
ISO 15985	Anaerobic digestion	Mineralization	T: 52 ± 2 °C Oxygen: anaerobic Inoculum from the operating anaerobic digester using high solids charge	Duration: minimum of 15 days Validity of test results: 70% biodegradation of the reference material	[75]

**Table 5:** Strategies to accelerate the biodegradation of biodegradable polymers based on UV treatments. Arrows (↑/↓) represent an increasing or decreasing performance for the selected indicator.

Feedstock	Valorization Strategy	Abiotic Suggested Parameters	Key Abiotic Degradation Indicators	Biodegradation Environment	Observation Reported	Ref.
PLA	UV irradiation + enzymatic degradation catalyzed with Proteinase K	UV-A light ***λ***: 300–700 nm I: 25.5 mW·cm^−2^ T: 45 °C RH: 65%, t: 60 h	Reduction in *M_n_*	Enzymatic hydrolysis: T: 37 °C t: 10–60 h pH: 8–8.6	Accelerated depolymerization after 60 h of irradiation	[161]
PLA	UV irradiation + *Stenotrophomonas* *maltophilia* LB 2–3	UV-C light ***λ***: 185–245 nm I: 6.41 × 10^−3^–3.22 mW·cm^−2^ t: 24 h	Reduction in *M_n_*, contact angle, and mechanical properties	Compost: T: 37 °C t: 24 h	Biodegradability increased after 8 h of UV-C irradiation, but became more resistant with longer exposure times	[169]
PLA	UV irradiation + ultrasonication + enzymatic degradation	UV light t: up to 6 h Ultrasonication 860 kHz	Surface oxidation, increased roughness and porosity	Enzymatic hydrolysis: T: 42 °C t: 8–16 weeks pH: 8.5	Up to 90% of mass loss after 16 weeks, and the hydrolysate was valorized to bacterial nanocellulose	[168]
PLA	UV irradiation + bioaugmentation + dairy wastewater sludge (*Pseudomonas geniculata* WS3)	UV-A-B-C light ***λ***: 340, 310, and 254 nm t: 150 min T: room temperature	↑ Brittleness Significant reduction in *M_n_* after 2 h of irradiation ↓*M_n/_M_w_*	Soil: T: 58 ± 2 °C RH: 40% pH: 4.3–7.9 Air flow: 25 mL·min^−1^	Enhanced weight loss (up to 90% in 12 days) and biodegradation in thermophilic conditions	[170]
PBAT/PLA blends	UV aging + microbial biodegradation (*Bacillus subtilis*)	I: 550 W·m^−2^, ***λ***: 300–800 nm t: 28 days T: 45 °C	*M_w_* reduction (31%). Carbonyl absorption bands decrease ↑ Roughness	Culture medium: T: 45 °C t: 35 days pH: 7.0	Biodegradation improved (62%) with a specific bacterial strain (*Bacillus subtilis*) and UV treatment	[149]
PBAT	UV irradiation + microbial biodegradation	UV-A light ***λ***: 320–400 nm t: 336 h I: 1.40 W·m^−2^·nm	Higher opacity and yellowish color, decrease in TS and ε, higher brittleness, increase in *E*, reduction in *M_w_*, crosslinking	Compost: t: 45 days	Photodegradation enhanced mineralization, only in the first stages, before crosslinking occurred after advanced irradiation	[90]
PLA, TPS, PBS, PBAT PLA/PBS, PLA/PBAT and PLA/TPS blends	UV aging + microbial biodegradation for base materials	Daylight filter and black standard ***λ***: 300–800 nm t: 21 days (6 months simulated conditions) T: 70 °C RH: water spraying	↑ Loss of color and whitening, especially for blends containing TPS and PBS Visible microcracks ↓ *TS* up to 87% with high TPS content	Compost: T: 58 ± 2 °C t: 65 days RH: water spraying	Under composting conditions, the neat polymers showed a high degree of disintegration, with TPS degrading the most rapidly	[117]

I stands for irradiation, ***λ*** for wavelength, T for temperature, RH for relative humidity, and t for time exposure conditions.

**Table 6:** Abiotic degradation of bioplastics through gamma and electron beam irradiation. Arrows (↑/↓) represent an increasing or decreasing performance for the selected indicator.

Feedstock	Ionizing Type	Abiotic Suggested Parameters	Key Abiotic Degradation Indicators	Observation Reported	Ref.
PVA/BC	Gamma (γ) irradiation	Source: ^137^Cs Dose: 20–50 kGy Dose rate: 0.4 kGy/h T: room temperature	↑ Yellowness/redness FTIR band shifts (-OH, C=O); chain scission at higher doses ↓ Crystallinity	BC is strongly affected by irradiation, confirmed by the splitting of the 1081 cm^−1^ band.	[173]
PHB/PEG	Gamma (γ) irradiation	Source: ^60^Co Dose: up to 40 kGy Dose rate: 5.72 kGy/h	↓ WVP (water vapor permeability) at >40 kGy ↓ Crystallinity	Loss of mechanical integrity at 40 kGy, with degradation noticed over 10 kGy.	[177]
PEC/PAAm	E-beam Irradiation	Source: E-beam accelerator, 3 MeV, 90 kW. Dose: 5–40 kGy	↑ Hydrophilicity ↓ Crystallinity Initial crosslinking (<10 kGy) and chain scission (40 kGy)	30 kGy resulted in optimal mechanical performance and stability, extended shelf-life.	[178]
Gelatin/glycerol	E-beam Irradiation	Source: E-beam accelerator, 2.2 MeV Dose: 20–60 kGy Dose rate: 0.3 kGy/s T: room temperature	↑ Hydrophilicity Crosslinking in the amorphous phase Formation of free radicals over >60 kGy dose	E-beam irradiation improves mechanical (↑ TS up to 30%) and surface functionality.	[179]
PLA	Gamma (γ) irradiation	Source: ^60^Co Dose: 30 kGy Dose rate: 3200 s/kGy T: room temperature	↓ *M_n_* and *M_w_* Free radical formation	Under simulated composting conditions (ISO 14855-1; 58 °C, for 60–141 days), both gamma- and E-beam-irradiated PLA reached ≥90% mineralization after 6 months of storage.	[180]
	E-beam Irradiation	Source: E-beam accelerator, 1.5 and 15 mA T: room temperature	↓ *M_n_*_/_*M_w_* Slight crosslinking		

**Table 7:** Strategies to accelerate the biodegradation of biodegradable polymers based on plasma treatment. Arrows (↑) represent an increasing performance for the selected indicator.

Feedstock	Valorization Strategy	Abiotic Suggested Parameters	Key Abiotic Degradation Indicators	Valorization Environment	Observation Reported	Ref.
PLA (89.5 kDa, 1 mm films)	Corona and RF plasma + accelerated weathering aging	Corona: *f*: 20.8 kHz t: 1 to 12 s	↑ Wettability Structural defects (voids, cracks, and cavities) ↑ Roughness ↑ *X_c_* before weathering, especially for RF plasma	UV/weathering aging: ASTM D4329 standard, radiation up to 2000 h	Degradation of PLA was more pronounced with RF than corona plasma pretreatment during weathering aging testing. Increase in hydrolytic degradation.	[191]
		RF: *f*: 13.56 MHz t: 15 to 180 s				
PLA-based cutlery items: cup strips (CSs) and spoon handles (SHs)	DBD-NTP + composting	V: 55, 75, and 100 V t: 20 and 80 min	↑ Roughness ↑ Wettability ↑ Carbonyl group index	Home-scale composting reactor: T: 50 ± 5 °C Air flow: 3 L·min^−1^	High voltage (HV) accelerated degradation rates, while low voltage and short duration enhanced oxidation. Complete disintegration within 20 days in CSs after HV.	[192]
Corn starch–PCL blends (30 µm thickness films)	RF-NTP+ soil	*f*: 13.56 MHz P: 40 W t: 0.5 to 5 min	↑ Roughness ↑ Wettability ↑ Mass loss for longer duration times	Soil: *Bacillus subtilis* MTCC 121. T = 30 °C. RH: 40–50%	Plasma preferentially affects the starch-rich domains within the blend. Modification of surface properties, enhancing adhesion and growth of BS 121.	[193]

**Table 8:** Strategies to accelerate the biodegradation of biodegradable and commodity polymers based on chemical and hydrolytic pretreatments. Arrows (↑) represent an increasing performance for the selected indicator.

Feedstock	Valorization Strategy	Abiotic Suggested Parameters	Key Abiotic Degradation Indicators	Biodegradation Environment	Observation Reported	Ref.
PLA PBAT PLA/PBAT/Starch	Thermo-alkaline + anaerobic degradation (wastewater treatment inoculum)	NaOH 1% (*w*/*v*) T: 70 °C t: 48 h	Pore structure formation Reduction in *M_w_*	Inoculum substrate ratio: 3 g VS/g VS t: 100 days	Methanogenic rate increases in the initial state, but MPs were found in the digestate.	[201]
PLA	Thermo-alkaline + anaerobic degradation	Ca (OH)_2_ 2% (*w*/*v*) T: 70 °C t: 48 h	↑ Roughness Structural defects (voids and cracks)	Inoculum substrate ratio: 2.85 g VS/g VS pH: 7–8 T: 38 °C t: 30 days	Biodegradation yield of 73%.	[148]
PET and PLA	DES pretreatment (dip-coating) + bacterial bioaugmentation	DES: choline chloride/glycerol 1:2 molar ratio Dip-coated—1 s Dried—1 h	↑ Surface wettability↑ Biofilm formation↑ Hydroxyl groups	Bacterial bioaugmentationAqueous mediumPilot-scale composting	DES enhanced microbial adhesion and composting degradation performance.	[120]
PLA	UV-C + DES + biosurfactant pretreatment + composting	ChCl/lactic acid (1:2); UV-C up to 8 h; composting 28 days	68.1% molar mass reduction↑ CO_2_ evolution	Composting and microbial biodegradation	DES enhanced the hydrophilicity and mineralization rate.	[203]
PET	Microwave-assisted DES pretreatment followed by enzymatic hydrolysis	Choline chloride/glycerol/ urea 1:2:1 molar ratio 260 W microwave, 3 min, 20 mL DES volume	16% monomer recovery↑ Carbonyl index and mass loss	Aqueous enzymatic system (LCC variant ICCG)	DES + microwave-enhanced enzymatic depolymerization.	[204]
PET	Anionic surfactants + enzymatic hydrolysis (PETase)	Bicine buffer pH 9.0, 30 °C; 500 nM PETase; surfactants: alkylsulfate, sulfonate, carboxylate	120× increase in PETase activity, 22% film thickness loss	Buffered aqueous enzymatic hydrolysis (PETase)	Surfactants improved enzyme alignment and activity.	[205]

**Table 9:** Strategies to accelerate the biodegradation of biodegradable polymers based on ozone treatments. Arrows (↑/↓) represent an increasing or decreasing performance for the selected indicator.

Feedstock	Valorization Strategy	Abiotic Suggested Parameters	Key Degradation Indicators	Valorization Environment	Observation Reported	Ref.
PHB	Ozone + biodegradation	t: 5 h Ambient conditions T: 24 °C RH: 35%	No impact on crystalline regions ↑ Tensile strength (25 MPa → 42 MPa); slight ↑ elongation at break (1%)	Soil: RH: 60% T: 22 ± 3 ^°^C pH: 6 t: 7, 28, 84, and 168 days	Slower biodegradation (300 days vs. 240 days for pristine PHB). Mass loss, volume loss, and surface defects (craters and cracks) after biodegradation.	[212]
Cassava starch (CS) PVA/NR/CS blends	Ozone + biodegradation	t: 50 min Ozone concentration: 20 mg·L^−1^ T: 50 °C	↑ Hydroxyl groups ↓Crystallinity ↑ Wettability	Soil: T: 27–28 ^°^C pH: 7 RH: 85% t: 30 days	The biodegradation rate of PVA/NR/CS blends increased in terms of mass loss (100% in 30 days with ≥15% of modified CS).	[214]

## Data Availability

Data sharing is not applicable in this study.

## List of Abbreviations and Symbols

AOPs	Advanced Oxidation Processes	PBSA	Poly(butylene succinate)-co-adipate
ATBC	Acetyl Tributyl Citrate	PCL	Poly(ε-caprolactone)
BC	Bacterial Cellulose	PE	Polyethylene
BMP	Biochemical Methane Potential	PEG	Poly(ethylene glycol)
BMPs	Biodegradable Microplastics	PET	Poly(ethylene terephthalate)
CL	Cellulose	Pec	Pectin
CT	Chitin	PHAs	Polyhydroxyalkanoates
CHT	Chitosan	PLA	Poly(lactic) acid
DES	Deep Eutectic Solvent	PP	Polypropylene
DSC	Differential Scanning Calorimetry	PS	Polystyrene
*E*	Tensile Modulus	PVA	Poly (vinyl alcohol)
***ε_B_***	Elongation to Break	PVC	Poly(vinyl chloride)
EPS	Extracellular Polymeric Substance	SRCs	Self-Reinforced Polymer Composites
FTIR	Fourier Transform Infrared Spectroscopy	*T_d_*	Decomposition Temperature
GHG	Greenhouse Gas	*T_g_*	Glass Transition Temperature
GPC	Gel Permeation Chromatography	TGA	Thermogravimetric Analysis
LDPE	Low-Density Polyethylene	*T_m_*	Melting Temperature
MCS	Modified Cassava Starch	TPS	Thermoplastic Starch
*M_n_*	Number-Average Molar Mass	*TS*	Tensile Strength
MPs	Microplastics	US	Ultrasonication
*M_w_*	Weight-Average Molar Mass	UV	Ultraviolet
NR	Natural Rubber	VS	Volatile Solids
PA	Polyamide	XRD	X-Ray Diffraction Spectroscopy
PAAM	Polyacrylamide	XRF	X-Ray Fluorescence
PBAT	Poly(butylene adipate)-co-terephthalate	XPS	X-Ray Photoelectron Spectroscopy
PBS	Poly(butylene succinate)		
